# Bio-interpretable ensemble learning model for invasive pulmonary adenocarcinoma grade using CT and histopathology images

**DOI:** 10.1038/s41698-025-01239-3

**Published:** 2025-12-18

**Authors:** Zhihe Yang, Fan Li, Qijia Han, Zhu Ai, Minyi Wu, Qiuxing Chen, Siqi Qu, Lingxiang Liu, Haowen Yan, Guorong Zou, Fang Chen, Hao Wang, Zhiming Xiang

**Affiliations:** 1 Cancer Institute of Panyu District, The Affiliated Panyu Central Hospital, Guangzhou Medical University, Guangzhou, PR China; 2 Department of Oncology, The Affiliated Panyu Central Hospital, Guangzhou Medical University, Guangzhou, PR China; 3 Department of Laboratory Medicine, Guangdong Provincial People’s Hospital Ganzhou Hospital, Ganzhou Municipal Hospital, Ganzhou, PR China; 4 Department of Radiology, The Affiliated Panyu Central Hospital, Guangzhou Medical University, Guangzhou, PR China; 5 Department of Pathology, The Affiliated Panyu Central Hospital, Guangzhou Medical University, Guangzhou, PR China

**Keywords:** Tumour biomarkers, Non-small-cell lung cancer, Computational biology and bioinformatics, Non-small-cell lung cancer, Cancer genomics, Cancer imaging, Cancer models, Tumour biomarkers, Tumour heterogeneity

## Abstract

The significant heterogeneity and complex morphology of invasive pulmonary adenocarcinoma (IPA) make grading challenging for pathologists. However, thorough investigations into radiopathomics features extracted from computed tomography (CT) and whole slide images (WSIs) for IPA grading and their biological significance remain limited. We aim to integrate multi-omics analysis to establish a robust grading model for IPA and reveal its biological significance. This multicenter study encompassed 988 patients who underwent radical surgical resection and received a pathological confirmation of IPA. Through integrated analysis of radiomics and pathomics, we constructed and validated an optimal ensemble learning grading model, which integrates multi-scale and multi-modal characteristics, achieved AUCs of 0.885, 0.920, 0.833, and 0.905 in the internal and external validation sets. Further systematic analysis of paired CT, WSIs, and RNA sequencing, two co-expression modules, 23 hub genes, and 680 significant pathways associated with grading were identified. Moreover, the reproducibility of the radiopathomics phenotypes, linked to multiple biological pathways—including signal transduction, cell differentiation, DNA damage and repair, cell proliferation and growth, metabolism, and metastasis and invasion—has been validated. In conclusion, the integration of radiological and pathological characteristics enhances the accuracy in differentiating high-grade IPA, offering a robust approach for grading. Multi-scale imaging biomarkers may promote personalized treatment.

## Introduction

Invasive pulmonary adenocarcinoma (IPA) is a more aggressive subtype of lung adenocarcinoma with a highly heterogeneous histologic pattern^1^. The novel grading system introduced by the International Association for the Study of Lung Cancer (IASCL), based on the histologic morphology of lung adenocarcinoma, is considered an important prognostic factor^2^. The high-grade IPA has a significantly poor prognosis and may benefit from adjuvant chemotherapy or immunotherapy^3–6^. Furthermore, it has been shown that this group is more prone to disease progression or recurrence after neoadjuvant therapy^7^. In this way, it is evident that accurate identification of the high-grade IPA is essential for patient prognosis and choice of treatment regimen. However, pathologists face numerous challenges in accurately identifying high-grade IPA. Approximately 80% of IPA contain two or more histological patterns, and pathologists need to estimate the percentage of each pattern in 5% increments during diagnostic grading, which is usually exceedingly time-consuming. In addition, the complexity of histological patterns, the high heterogeneity of tumors, and the subjective factors of pathologists challenge the consistency of diagnostic results tremendously^8^. Therefore, it is urgent to develop an efficient and reproducible method to identify patients in high-grade IPA.

Artificial intelligence (AI) has made significant advancements in the medical field in recent years. It utilizes multilayer feature extraction and large-scale data training to capture complex characteristics in medical images and reveal hidden patterns in medical data^9^, particularly in cancer research^10,11^. The extracted features from medical images fall into two main categories: handcrafted features, which are manually designed and extracted by human experts using specific algorithms and domain knowledge, and deep learning (DL) features, which are automatically identified through multilayer neural networks^12–14^. These two types of features are considered complementary^14^. Additionally, with the progress of digital pathology technology, tasks related to tumor grading and prognosis based on digital pathology images are gradually emerging^15,16^. Integrating multimodal and multiscale data has been shown to more comprehensively reflect tumor heterogeneity and improve the predictive performance of models^17–19^. However, the potential value of combining macro- and micro-level image features for the grading of IPA requires further exploration. Moreover, interpretability is paramount in clinical decision-making, particularly in high-stakes treatment determinations^20,21^. While machine learning models leveraging extensive training data can unveil data patterns, they often overlook pertinent prior knowledge in the domain of biology, which is essential for comprehending disease progression. The lack of biological interpretability is a notable barrier impeding the acceptance of AI models within the medical realm^22^. To our knowledge, there is limited research on the biological interpretability and reproducibility of radiopathomics features for IPA grading.

Here, we propose an ensemble learning model for IPA grade by integrating DL and handcrafted radiopathomics features (DHRPs). Subsequently, we elucidated the biological underpinnings of these features.

## Results

### Study design and patient characteristics

**Fig. 1:**
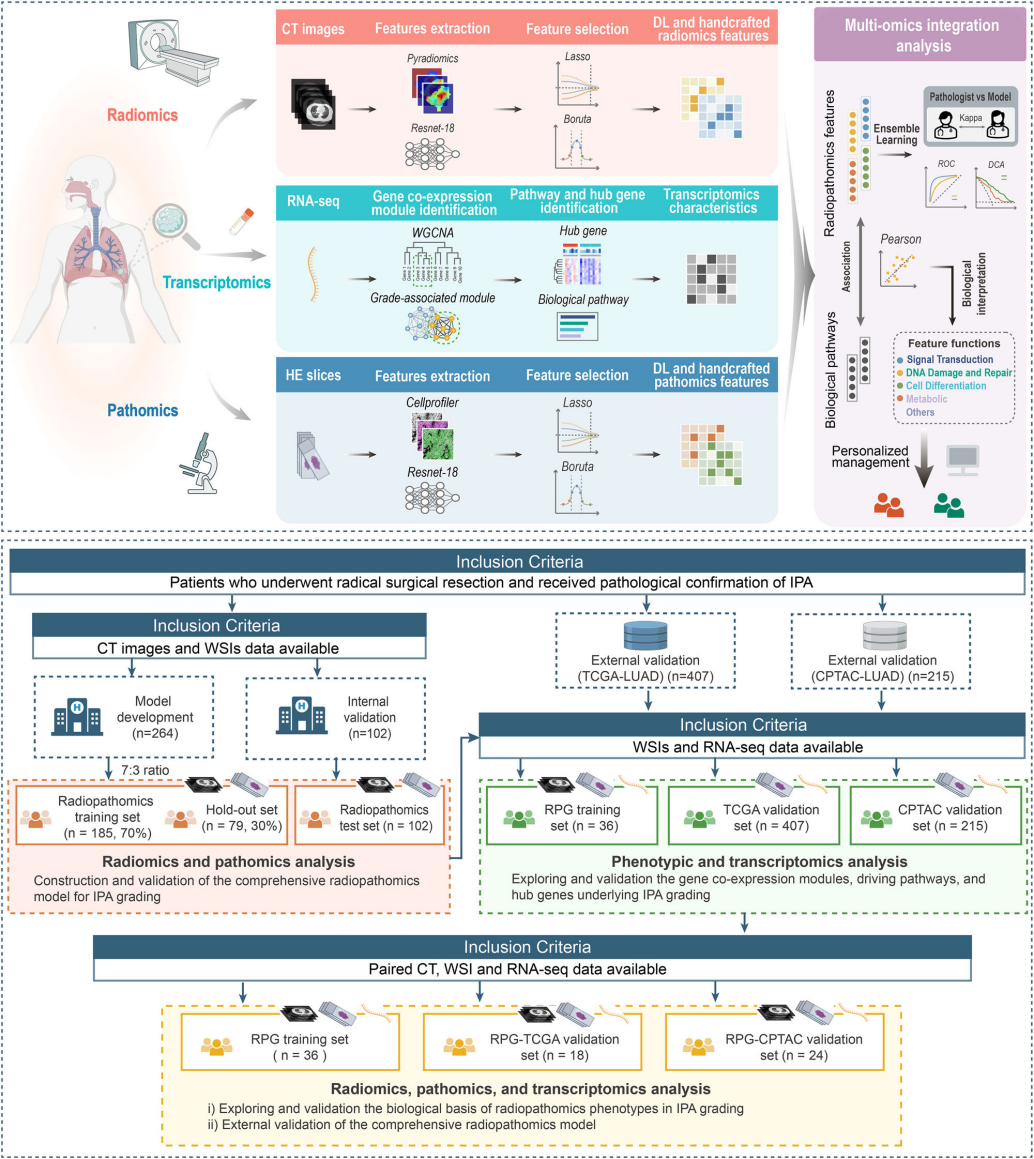
Study design and patient enrollment. The workflow includes three key areas: (1) acquisition and analysis of both handcrafted and deep learning (DL) features from radiological images (CT scans); (2) acquisition and analysis of features from pathological images (WSIs); and (3) identification of grading-related gene co-expression modules, hub genes and exploration of associated biological pathways. Multi-modal data integration subsequently facilitated the construction of a comprehensive grading model for IPA, enabling the exploration of the potential biological functions of macro- and micro-image features. (Some elements in Fig. 1 were adapted from templates from BioRender.com). TCGA The Cancer Genome Atlas, CPTAC Clinical Proteomic Tumor Analysis Consortium, DHRPs deep learning and handcrafted radiopathomics models, RPG Rad-Pat-Genomics, WSIs whole slide images, DL deep learning, IPA invasive pulmonary adenocarcinoma, ROC receiver operating characteristic, DCA decision curve analysis.

The overall study design and patient enrollment process are illustrated in Fig. 1. Patient data were obtained from our institution and two public datasets (TCGA-LUAD and CPTAC-LUAD datasets). A total of 988 patients with IPA from four datasets (Radiopathomics analysis set, *n* = 264; Radiopathomics test set, *n* = 102; TCGA validation set, *n* = 407; CPTAC validation set, *n* = 215) were included in this study (Table S1, Supporting Information). Notably, to develop the radiopathomics grading model, the radiopathomics analysis set was randomly divided into the radiopathomics training set (*n* = 185) and hold-out set (*n* = 79) in a 7:3 ratio for model development and validation (Table S2, Supporting Information). Additionally, to analyze the biological basis underlying grading phenotypes in IPA, three sub-datasets were selected: (i) Rad-Pat-Genomics (RPG) training set (*n* = 36); (ii) RPG-TCGA validation set (*n* = 18); (iii) RPG-CPTAC validation set (*n* = 24), with detailed information available in Table S3 (Supporting Information). The RPG training set containing paired WSI and RNA data, along with the TCGA validation set and the CPTAC validation set, were used to develop and validate grade-associated gene co-expression modules and hub genes. In the RPG-TCGA and RPG-CPTAC validation sets, CT and WSIs data were used to validate the models externally, while paired CT, WSIs, and RNA sequencing data confirmed feature-biological function associations.

### Feature selection, and visualization

**Fig. 2:**
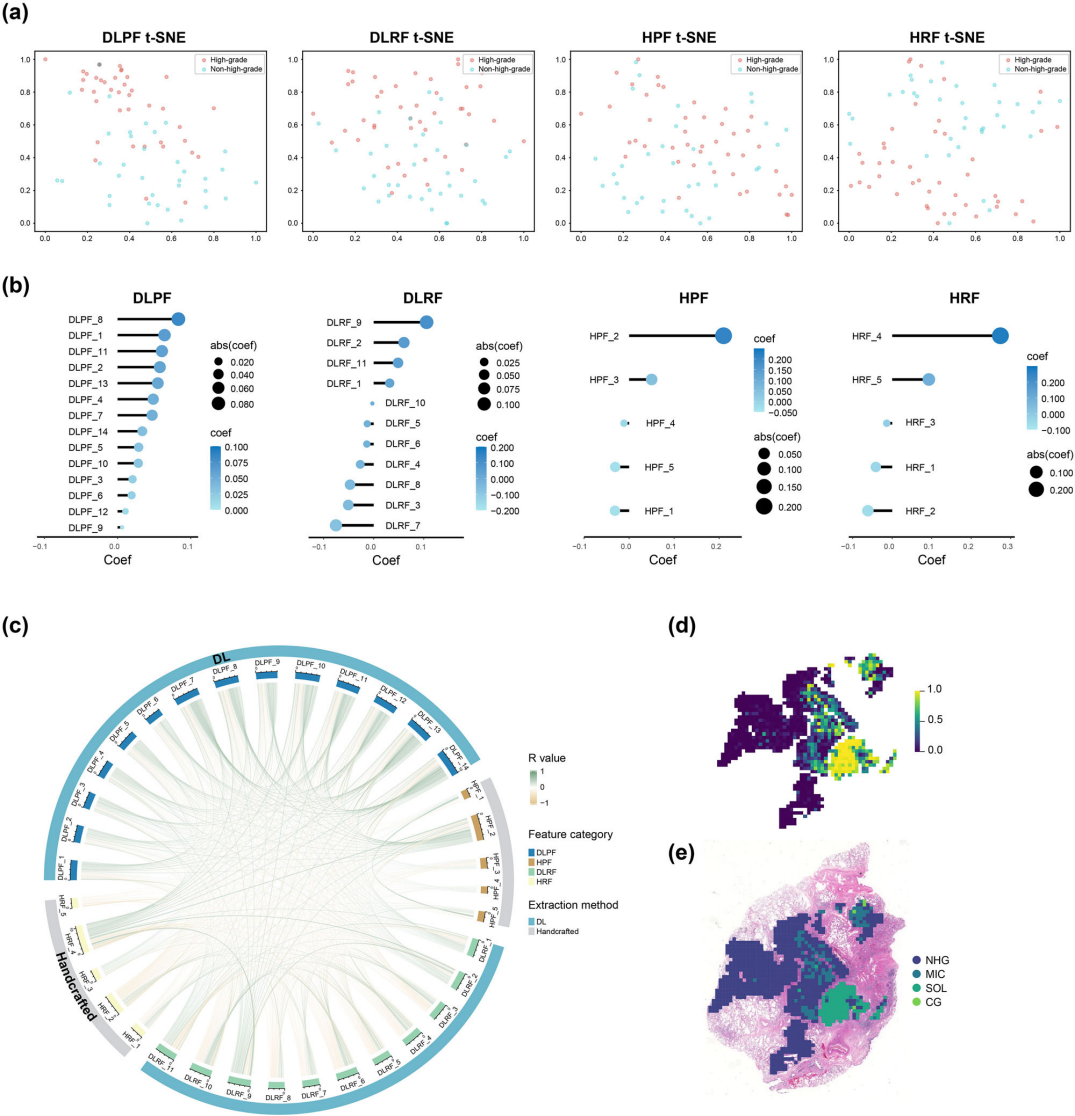
Discriminative ability, coefficient weight, and correlations of deep learning and handcrafted radiopathomics features. **a** T-distributed stochastic neighbor embedding (t-SNE) visualizations of deep learning and handcrafted radiopathomics features in the hold-out set, with red dots indicating high-grade IPA and blue dots representing non-high-grade IPA. **b** Visualization of the coefficients of the top four types of features retained after LASSO feature selection. **c** The chord diagram shows intergroup Spearman correlations among the top four features, with the color of the connections indicating the magnitude of the correlation coefficient. Visualization of the patch-level prediction process: The upper image presents the predicted probability for each patch **d**, while the lower image illustrates the prediction results for each patch within the WSI **e**. LASSO, least absolute shrinkage and selection operator. NHG non-high-grade, MIC micropapillary, SOL solid, CG complex gland.

Independent feature selection processes were applied to the handcrafted radiomics features (HRF), DL radiomics features (DLRF), HRF, and DLRF, ultimately retaining 5, 11, 5, and 14 optimal features, respectively. T-SNE dimension reduction was used for the visualization of these four category features (Fig. 2a). Furthermore, the weights of the feature coefficients retained after least absolute shrinkage and selection operator (LASSO) screening for each category of features are presented in Fig. 2b and Table S4 (Supporting Information). The Spearman correlation analysis showed that there was generally a low correlation between the features within each category of features (Fig. S1), except for the correlation between DLPF_14 and DLPF_13, which had a relatively higher R-value of 0.669. There was also no strong correlation between the different categories of features, with a correlation coefficient of less than 0.6 (Fig. 2c). To assess inter-institutional stability, we conducted a signal-to-noise ratio (SNR) analysis on the final feature set used for model construction (Fig. S2). All handcrafted features (HPF and HRF) demonstrated excellent robustness (SNR ≥ 2). Although a subset of DL–derived features exhibited moderate stability (SNR between 1 and 2), their reliability remained acceptable. The relatively higher noise observed in DL features is likely attributable to their high-dimensional and abstract representations, which may be more sensitive to, and thus amplify, technical variations (Fig. S3).

It is noteworthy that in the domain of DLPF, the process of feature extraction involves the utilization of the Multiple Instance Learning (MIL) paradigm. This methodology encompasses the initial generation of predictions at the patch level, followed by the aggregation of prediction probabilities from all patches of each patient into a WSI-level feature representation. This representation assigns higher weights to key patches. The heatmap (Fig. 2d, e) visually presents the patch-level prediction probabilities and the patch predictions within the WSIs for high-grade IPA.

### Model performance

**Fig. 3:**
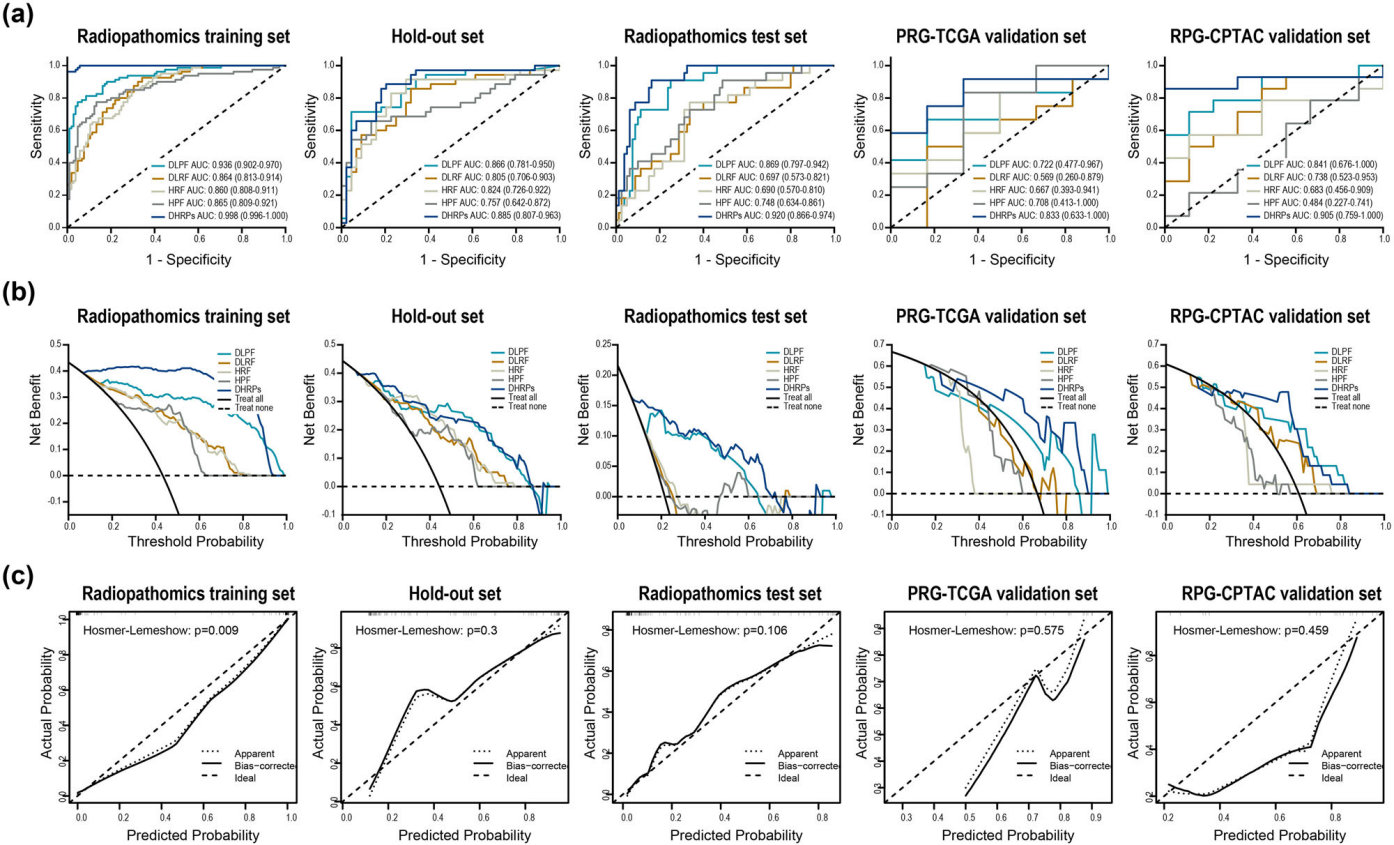
Performance of DL and handcrafted radiopathomics features. **a** Comparison of ROC curves for unimodal features and combined radiopathomics features across multiple sets: the radiopathomics training set, hold-out set, radiopathomics test set, RPG-TCGA validation set, and RPG-CPTAC validation set. **b** Comparison of DCA curves for different models. **c** Calibration curves of the DHRPs model across various datasets. ROC receiver operating characteristic, DCA decision curve analysis.

The performance metrics for each model within each dataset are presented in Fig. 3 and Table 1. In the radiopathomics training set, the AUCs for the four modalities (DLPF, HRF, HPF, and DLRF) were 0.936 (0.902–0.970), 0.860 (0.808–0.911), 0.865 (0.809–0.921), and 0.864 (0.813–0.914), respectively. The DHRPs model had an AUC of 0.982 (0.969–0.995), which was superior to all single-modality models (DeLong test, *P* < 0.05). The DHRPs model achieved the highest sensitivity and accuracy (0.925 and 0.930, respectively), with the sensitivity of the DHRPs model (0.925) being sufficiently high. The models were validated using the hold-out set, radiopathomics test set, RPG-TCGA validation set, and RPG-CPTAC validation set. The DHRPs model achieved the highest AUC in all validation sets, with AUCs of 0.885 (0.807–0.963) in the hold-out set, 0.920 (0.866–0.974) in the radiopathomics test set, 0.833 (0.633–1.000) in RPG-TCGA validation set, and 0.905 (0.759–1.000) in RPG-CPTAC validation set. The DLPF model performed well in the hold-out set, radiopathomics test set, and RPG-CPTAC validation set, with AUC values of 0.866 (0.781–0.950), 0.869 (0.797–0.942), and 0.841 (0.676–1.000), respectively. However, its performance in the RPG-TCGA validation set was moderate, with an AUC of 0.722 (0.477–0.967). The HPF model generally showed poor to moderate performance across all validation sets, with AUCs of 0.757 (0.642–0.872) in the hold-out set, 0.748 (0.634–0.861) in the radiopathomics test set, 0.708 (0.413–1.000) in RPG-TCGA validation set, and 0.484 (0.227–0.741) in RPG-CPTAC validation set. The HRF model performed well in the hold-out set and radiopathomics test set, with AUCs of 0.824 (0.726–0.922) and 0.845 (0.757–0.934), respectively. However, in RPG-TCGA and RPG-CPTAC validation sets, their performance was moderate, with AUCs of 0.667 (0.393–0.941) and 0.683 (0.456–0.909). The DLRF performed well in the hold-out set with an AUC of 0.805 (0.706–0.903). However, their performance was moderate in the radiopathomics test set, RPG-TCGA validation set, and RPG-CPTAC validation set, with AUCs of 0.697 (0.573–0.821), 0.569 (0.260–0.879), and 0.738 (0.523–0.953), respectively. The DHPRs model exhibited superior clinical net benefit across most threshold ranges (Fig. 3b). The calibration curves (Fig. 3c) illustrate the concordance between the predicted and actual observed values of the DHRPs model within each dataset.

**Table 1:** Performance metrics for unimodal as well as comprehensive models across datasets

Datasets and metric	Feature type	AUC (95% CI)	Accuracy	Sensitivity	Specificity	PPV	NPV
Radiopathomics training set	DLPF	0.936 (0.902–0.970)	0.870	0.763	0.952	0.924	0.840
	HRF	0.860 (0.808–0.911)	0.762	0.838	0.705	0.684	0.851
	HPF	0.865 (0.809–0.921)	0.822	0.763	0.867	0.813	0.827
	DLRF	0.864 (0.813–0.914)	0.768	0.913	0.657	0.670	0.908
	DHRPs	0.982 (0.969–0.995)	0.930	0.925	0.933	0.914	0.942
Hold-out set	DLPF	0.866 (0.781–0.950)	0.835	0.686	0.955	0.923	0.792
	HRF	0.824 (0.726–0.922)	0.785	0.886	0.705	0.705	0.886
	HPF	0.757 (0.642–0.872)	0.759	0.629	0.864	0.786	0.745
	DLRF	0.805 (0.706–0.903)	0.747	0.829	0.682	0.674	0.833
	DHRPs	0.885 (0.807–0.963)	0.823	0.857	0.795	0.769	0.875
Radiopathomics test set	DLPF	0.869 (0.797–0.942)	0.775	0.864	0.750	0.487	0.952
	HRF	0.845 (0.757–0.934)	0.735	0.864	0.700	0.442	0.949
	HPF	0.748 (0.634–0.861)	0.588	0.864	0.513	0.328	0.932
	DLRF	0.697(0.573–0.821)	0.667	0.682	0.663	0.357	0.883
	DHRPs	0.920 (0.866–0.974)	0.843	0.864	0.838	0.594	0.957
RPG-TCGA validation set	DLPF	0.722 (0.477–0.967)	0.667	0.583	0.833	0.875	0.500
	HRF	0.667 (0.393–0.941)	0.667	0.750	0.500	0.750	0.500
	HPF	0.708 (0.413–1.000)	0.722	0.750	0.667	0.818	0.571
	DLRF	0.569 (0.260–0.879)	0.556	0.417	0.833	0.833	0.417
	DHRPs	0.833 (0.633–1.000)	0.667	0.500	1.000	1.000	0.500
RPG-CPTAC validation set	DLPF	0.841 (0.676–1.000)	0.739	0.643	0.889	0.900	0.615
	HRF	0.683 (0.456–0.909)	0.652	0.500	0.889	0.875	0.533
	HPF	0.484 (0.227–0.741)	0.565	0.714	0.333	0.625	0.429
	DLRF	0.738 (0.523–0.953)	0.696	0.786	0.556	0.733	0.625
	DHRPs	0.905 (0.759–1.000)	0.870	0.786	1.000	1.000	0.750

*DLPF* deep learning pathomics features, *DLRF* deep learning radiomics features, *HRF* handcrafted radiomics features, *HPF* handcrafted pathomics features, *DHRPs* deep learning and handcrafted radiopathomics features, *AUC* area under the curve, *CI* confidence interval, *PPV* positive predictive value, *NPV* negative predictive value.

### Comparative evaluation of diagnostic performance between the model and pathologists

As shown in Figure S4, the operating points of both pathologists fall in the lower-right region of the DHRPs model ROC curve. Operating point matching analysis (Table S5) indicated that both pathologists exhibited lower sensitivity than the model (although differences were not statistically significant), while the model achieved significantly higher specificity compared with one pathologist (*p* < 0.05), indicating that the model performs comparably to or even better than board-certified pathologists with limited clinical experience in discriminatory ability. Furthermore, substantial variation was observed in inter-observer agreement across histological subtypes: IASLC grading (*κ* = 0.744, substantial), solid subtype (*κ* = 0.756, substantial), papillary subtype (*κ* = 0.414, moderate), micropapillary subtype (κ = 0.143, poor), lepidic subtype (*κ* = 0.382, fair), complex glandular subtype (*κ* = 0.476, moderate), and acinar subtype (*κ* = 0.561, moderate). These findings highlight marked inter-observer variability in conventional pathological diagnosis, particularly in subtypes such as the micropapillary pattern, where subjective differences are pronounced.

### Gene co-expression module identification, module preservation analysis, and module-trait correlation

**Fig. 4:**
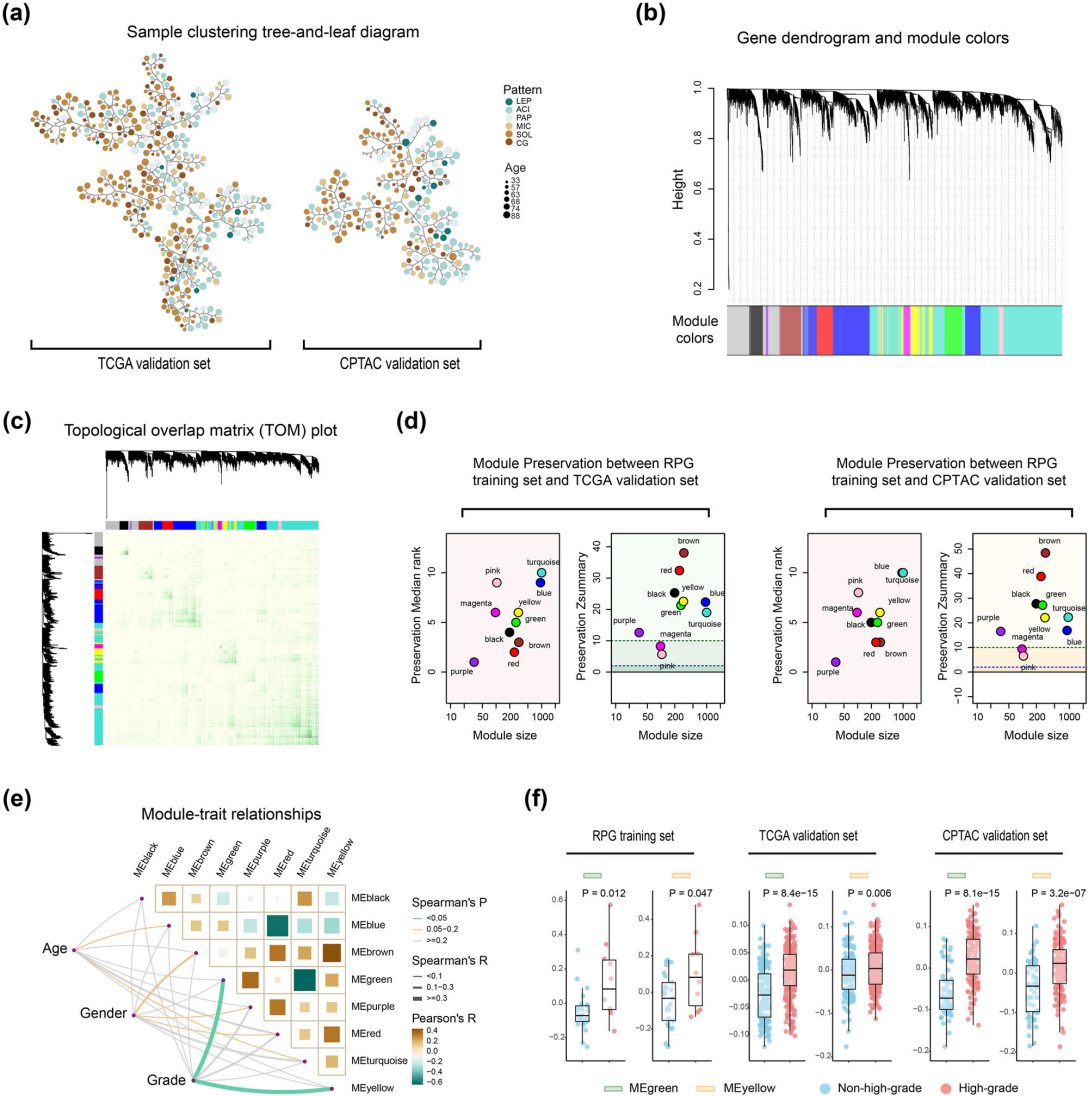
WGCNA and identification of grading-related modules. **a** Clustering tree-and-leaf diagram of samples in the TCGA validation set and CPTAC validation set. Genes were clustered, and patient phenotypes were visualized using tree and force-directed layout algorithms. Samples were divided into several main branches, with each leaf representing a patient. The size and color of the leaves indicate the patient’s age and primary growth pattern, respectively. **b** Clustering dendrogram of gene co-expression modules in the RPG training set. **c** Heatmap of correlations among genes. Rows and columns represent genes, with different colors indicating different modules. The internal heatmap colors represent the interaction strength between genes, with stronger interactions shown in green. Weaker interactions between modules suggest high independence among the modules. **d** Zsummary and median rank statistics for module preservation analysis between the RPG training set and the TCGA validation set, as well as between the RPG training set and the CPTAC validation set. Different colors represent different gene modules, with the green horizontal dashed line indicating a Zsummary value of 10, and the blue dashed line indicating a value of 2. **e** Correlation among modules and the association between modules and phenotypes in the RPG training set. **f** Boxplot displaying the differences in expression levels of module eigengenes within the green and yellow modules across different grading groups in the RPG training set, TCGA validation set, and CPTAC validation set. The horizontal axis represents different grading groups, and the vertical axis represents the expression levels of module eigengenes. A *p* < 0.05 indicates a significant difference. WGCNA Gene co-expression network analysis.

The Weighted Gene Co-expression Network Analysis (WGCNA) results are shown in Fig. 4a–c and Fig. S5 (Supporting Information). Based on the RPG training set, eleven co-expression modules were derived. Figure 4d and Tables S6 and S7 (Supporting Information) present the module preservation analysis results. In the TCGA and CPTAC validation sets, eight modules with Zsummary ≥10 demonstrated strong evidence of preservation, and the detailed descriptions are provided in Fig. S6 (Supporting Information).

Notably, the green (273 genes) and yellow (309 genes) modules, detailed in Table S8 (Supporting Information), consistently correlated with IPA grading across three datasets (RPG training set, TCGA validation set, and CPTAC validation set). This correlation was evidenced by significant Spearman correlations (green module: *r* = 0.42, 0.39, 0.54; yellow module: *r* = 0.34, 0.14, 0.35; all *P* < 0.05), as shown in Fig. 4e and Fig. S7 (Supporting Information). Our post-hoc analysis confirms that the sample size was overall sufficient to robustly detect module-phenotype associations, despite reduced power for weaker correlations in the smallest dataset (Table S9). Furthermore, Fig. 4f displays the differences in expression levels of module eigengenes within the green and yellow modules. The two modules, demonstrating stability and significance, facilitated the investigation into grading-associated biological pathways.

### Exploring the driving pathways and hub genes underlying IPA grading

In the RPG training set, the green and yellow modules collectively enriched 892 pathways (FDR < 0.05). Similarly, pathway enrichment analysis was conducted on these two modules in the external validation sets, and the results show that most of the pathways remained enriched (Fig. S8a–c, Supporting Information). In the green module, 313 pathways (14 negatively and 299 positively correlated) remained enriched in the TCGA and CPTAC validation sets. In the yellow module, 367 pathways (1 negatively and 366 positively correlated) remained enriched. The top 5 enriched pathways are listed in Tables S10–S12 (Supporting Information). All pathways information can be found at https://github.com/holsenyang/Radio-Patho-Genomics.

Figure S8d (Supporting Information) exhibits the two top pathways consistently upregulated and downregulated in the Gene Set Enrichment Analysis (GSEA)^23^ across the TCGA and CPTAC validation sets. Genes in cell cycle-related pathways (cell cycle and mitotic cell cycle) showed a consistent upregulation trend in high-grade IPA, indicating active cell proliferation in this group. Conversely, activity in specific transmembrane transport and endosomal functions exhibited a significant downregulation trend, suggesting alterations affect tumor cells’ migratory and invasive abilities. Table S13 (Supporting Information) lists the top 10 positively correlated and top 10 negatively correlated pathways from the GSEA in the TCGA and CPTAC validation sets.

The green and yellow modules identified 23 hub genes associated with IPA grading (Table S14, Supporting Information). The selection of reproducible hub genes between the RPG training set and the CPTAC validation set is presented in Fig. S9a–d and Fig. S11a (Supporting Information), and the reproducibility of hub genes between the RPG training set and the TCGA validation set is shown in Fig. S10a–d and Fig. S11a (Supporting Information). The expression levels of hub genes in the RPG training set, TCGA validation set, and CPTAC validation set are illustrated in Fig. S11b, c and Fig. S9e (Supporting Information).

### Biological interpretation of radiopathomics phenotypes in IPA grading

**Fig. 5:**
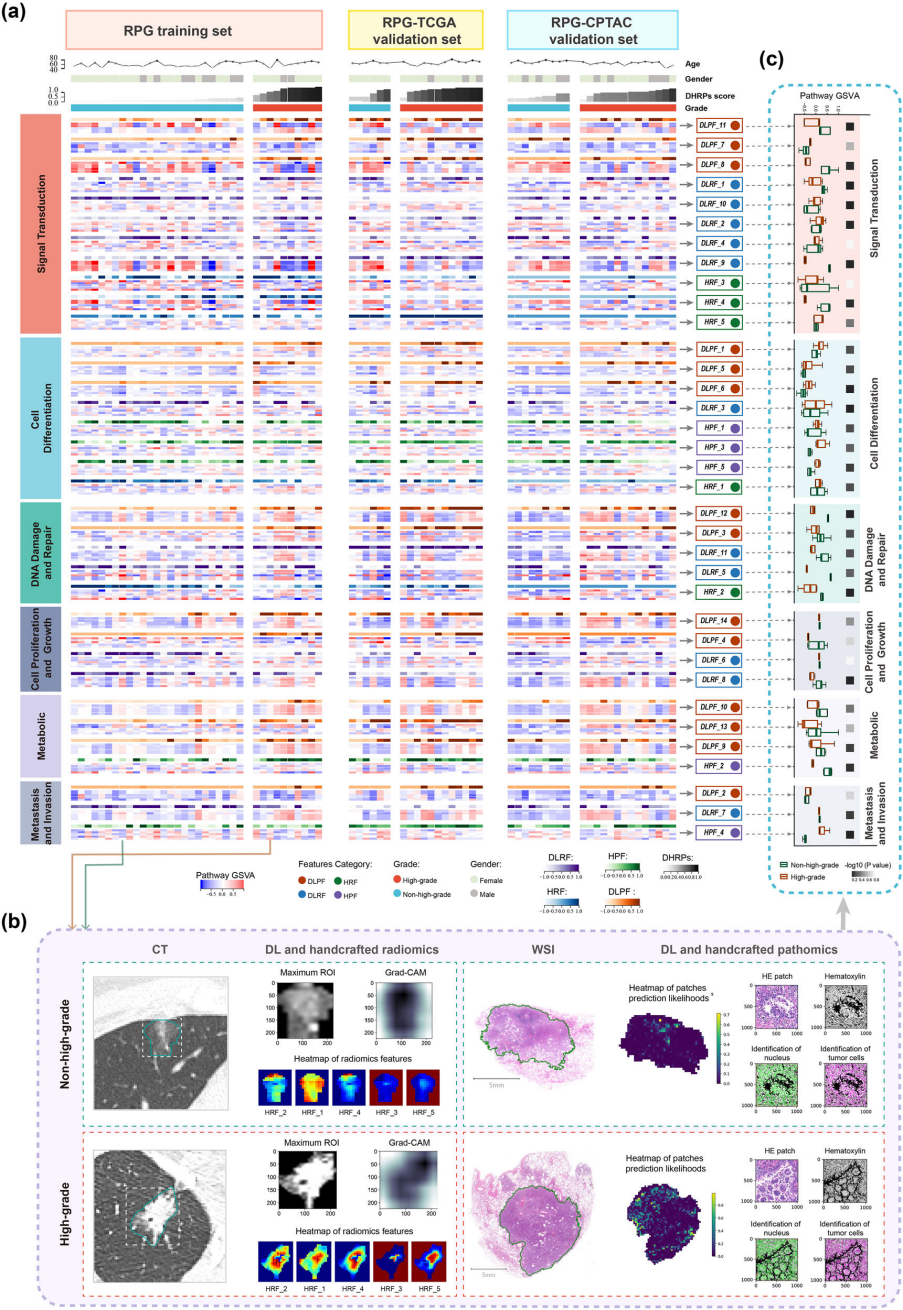
Association between DL and handcrafted radiopathomics features and significantly enriched pathways. **a** Heatmap shows the 35 retained radiopathomics features (11 DL radiomics features, 5 handcrafted radiomics features, 5 handcrafted pathomics features, and 14 DL pathomics features) and their top 5 correlated pathways, represented by their gene set variation analysis (GSVA) scores. The five rows following each image feature indicate the activation levels of the top five significant pathways. Additionally, differences among patient subgroups in the RPG training set, RPG-TCGA validation set, and RPG-CPTAC validation set, as well as their age, gender, and DHRPs model prediction scores, are displayed. **b** This panel highlights the distinctions observed in CT and WSIs of two patients diagnosed with high-grade IPA and non-high-grade IPA. The left section displays the focus areas of the DL model during feature extraction, visualized using Grad-CAM, where darker shades signify greater model emphasis. Additionally, the heatmap variations of handcrafted radiomics features (HRF_1 to HRF_5) are also depicted. The right side shows differences in parameters within the corresponding WSIs of the two patients, with a probability prediction heatmap indicating the predicted probabilities of all patches in the DLPF model. Furthermore, during handcrafted pathomics feature extraction, segmentation maps of tumor cells and nuclei within the patches (acinar pattern in non-high-grade and micropapillary pattern in high-grade) are shown, with handcrafted pathomics features derived from the identified nuclei and cell analyses. **c** Grouped box plots depict differences in the top 5 pathways most associated with each image feature in these two patients.

We investigated the enriched pathways within the selected modules and their association with each radiopathomics feature to elucidate their biological significance (Table S15, Supporting Information). Radiopathomics phenotypes can be classified into six primary categories: signal transduction, cell differentiation, DNA damage and repair, cell proliferation and growth, metabolism, and metastasis and invasion. Correlation heatmaps displaying the radiopathomics grading phenotypes and their relevant pathways for each patient in the RPG training set, TCGA validation set, and CPTAC validation set are depicted in Fig. 5a. Furthermore, the differences captured from CT images and WSIs of two patients with high- and non-high-grade IPA are illustrated in Fig. 5b. Grouped boxplots (Fig. 5c) illustrate the differences in the five pathways most strongly linked to each image feature between the two patients. Figure S12 and Table S16 present the statistical power distribution of the feature-pathway associations and the corresponding Minimum Detectable Correlation Coefficient (MDCC) under the current sample size.

## Discussion

The IASLC grading system has emerged as a practical prognostic stratification tool for IPA in clinical settings^2,24^. Nevertheless, the intricate morphology and high intratumoral heterogeneity present notable challenges for pathologists in the grading process. While AI models based on medical imaging hold promise for automating cancer grading^25–27^, the biological significance of these imaging features largely remains unknown. In this investigation, we developed and validated a bio-interpretable comprehensive radiopathomics model for IPA grading.

In recent years, several studies have demonstrated the value of radiomics in identifying histological subtypes of lung adenocarcinoma^28–30^. However, these studies did not consider the proportion of high-grade subtypes or the complex glandular patterns. In a preliminary investigation, we verified the predictive value of CT radiomics for high-grade IPA^31^. However, it should be noted that these radiological image-based investigations provide mainly a macroscopic overview of the tumor’s histological patterns, serving as an indirect examination method. In contrast, WSIs may capture subtle variations in tumor cells or tissues at the micro-level directly, as evidenced by studies proving the value of pathological image characteristics for cancer prognosis and grading^13,32^. Indeed, a recent study indicates that incorporating multi-scale and multi-modal information might boost model predictive ability. These macroscopic and microscopic features appear to complement each other, offering a more comprehensive depiction of tumor heterogeneity^19,33,34^. Additionally, the combination of handcrafted and DL features can further improve model performance^35–37^. Our study integrated DL and handcrafted features from CT and WSIs, ultimately achieving optimal performance. Nevertheless, the model exhibited suboptimal calibration, which may be attributed to the integration of 11 base learners; calibration disparities and feature dependencies among these models might have reduced ensemble diversity and amplified bias, thereby impairing overall calibration performance. In contrast to the time-consuming traditional approach that relies on manual microscopic evaluation, our model achieves comparable or even superior discriminatory performance relative to board-certified pathologists while reducing the entire diagnostic process to a matter of minutes following feature extraction from large-scale imaging data. Furthermore, Kappa analysis in this study revealed that although substantial agreement between the two pathologists was observed in IASLC grading, considerable subjectivity remained in the assessment of certain IPA subtypes—particularly micropapillary and lepidic patterns. Such variability likely arises from morphological overlap between lepidic and acinar patterns due to alveolar collapse, evolving diagnostic criteria (e.g., filamentous micropapillary forms), and the interpretive challenges posed by tumor cell detachment. By leveraging objectively quantifiable features, our model provides a stable and reproducible diagnostic benchmark, thereby mitigating inter-observer variability and enhancing diagnostic consistency and reliability.

In our investigation of CT images, we have identified that the Small_Area_High_Gray_Level_Emphasis texture feature from the gray-level size zone matrix (GLSZM) and the minimum pixel intensity from first-order features are closely linked to high-grade IPA. The minimum pixel intensity accentuates the opaque ground-glass tumor areas, which aligns with the findings of Song^28^, suggesting that the minimum pixel value across the entire image could predict micropapillary components. The Small_Area_High_Gray_Level_Emphasis feature suggests the presence of smaller and denser high-gray-level areas in high-grade IPA images, potentially indicating other high-grade components. Furthermore, in the context of DLRF, the Grad-CAM has elucidated that the model assigns significant weights to diverse regions in high-grade IPA and non-high-grade IPA. Concerning WSIs, our scrutiny reveals that the Zernike shape features from cells, the location of maximum intensity, and the entropy of texture features play pivotal roles in grading. Prior research by Yu et al.^38^ has also demonstrated the relevance of shape features of tumor cell nuclei and cytoplasm and nuclear texture features in lung adenocarcinoma prognosis. Notably, our adoption of the MIL paradigm in the DLPF model involved the formation of patch-level predictions. This process, as indicated by Grad-CAM, effectively captured differences in growth patterns. Subsequently, aggregating patch prediction results into WSI-level feature representations ensured that key patches were afforded higher weights.

Although machine learning and DL have shown tremendous potential in the medical field, most studies rely solely on data-driven approaches, neglecting the biological explanations of diseases. Exciting new research is delving into the biological implications of imaging characteristics. For instance, in a study by Grossmann et al.^39^, a correlation was established between imaging features, immune response, inflammation, and overall survival. What’s more, characteristics of intra-tumor heterogeneity could anticipate RNA polymerase transcription activity and intensity dispersion, which could predict the autodegradation pathway of a ubiquitin ligase. In another study, Cao et al.^40^ identified DL features from digital pathology images associated with anti-tumor immune activation pathways. However, many previous studies exploring the biological significance of imaging features focused solely on differential genes^41,42^, disregarding the interactions between genes. Furthermore, few studies comprehensively investigate the biological mechanisms of DL and handcrafted imaging features. In contrast, our study used the WGCNA method to identify interacting modules. Then, it explored the biological pathways of the radiopathomics features within grade-related modules, enhancing interpretability and reproducibility. This approach is similar to several previous studies of the identification of gene modules in breast cancer, glioblastoma, and clear cell renal cell carcinoma^43–45^. Our findings indicate a significant association of genes in the green and yellow co-expression modules with high-grade IPA. We acknowledge that the correlation of the yellow module in the smallest subset (*n* = 36, power = 0.539) should be interpreted as preliminary and hypothesis-generating. However, the detection of a stronger effect (*r* = 0.42, power = 0.740) for the green module in the same subset, along with analyses in the external datasets (*n* = 407 and *n* = 215) achieving near-100% power, robustly supports the overall validity of our findings and the exploratory nature of this study. Furthermore, pathways enriched in these modules demonstrate robust replication in the external validation sets.Genes associated with cell cycle pathways displayed a consistent up-regulation trend in high-grade IPA, potentially indicating heightened cell proliferation compared to the non-high-grade IPA. Conversely, activities related to specific transmembrane transporter and endosomal functions were significantly down-regulated. These changes may impact the migratory and invasive capabilities of tumor cells.

Radiopathomics features associated with IPA grading primarily map to six distinct biological processes. CT-based features are mainly associated with functions such as signal transduction and cell differentiation. This correlation reflects an intrinsic biological mechanism: CT-derived macroscopic heterogeneity non-invasively mirrors underlying microscopic cellular and structural variation within tumors^46^. This structural heterogeneity is driven largely by spatial disparities in signaling pathway activity and cancer cell–tumor microenvironment (TME) interactions. In lung adenocarcinoma, aberrant activation of pathways such as MAPK/ERK promotes proliferation, invasion, and metastasis^47^. Such activation is spatially heterogeneous—for example, cells at the invasive front with elevated ERK signaling may form dense clusters and fibrotic reactions that appear on CT as spiculation or pleural retraction^46,48^. Thus, radiologically heterogeneous tumors likely harbor dysregulated cellular signaling, transcriptomically reflected as enriched signal transduction pathways. These findings suggest that quantitative CT features may serve as non-invasive biomarkers for dynamically monitoring tumor biological states during treatment. Conversely, pathomics features from WSIs showed broader associations with metabolic pathways and other biological processes. WSIs offer the advantage of capturing fine-grained morphological phenotypes of tumor cells and their interactions with diverse TME components—immune cells, fibroblasts, vascular endothelial cells—with subcellular resolution. Metabolic reprogramming drives these cellular behaviors and tissue remodeling. Metabolic reprogramming serves as the intrinsic force driving these cellular behaviors and tissue architectural changes. Consequently, WSI analysis enables high-throughput quantification of subtle morphological patterns, thereby indirectly decoding the underlying highly active metabolic functional states. Overall, diverse histological patterns in tumors arise from distinct internal biological processes, ultimately producing heterogeneous imaging phenotypes. Comprehensive profiling of high-grade IPA imaging features, coupled with mechanistic insights, is essential for advancing precision oncology.

Our study has several limitations. Firstly, the improved performance of the DHRPs model in external validations may be due to the limited sample size and selection bias; hence, larger-scale studies are needed for future validation. Secondly, disparities in CT scan parameters among institutions may lead to data heterogeneity and uncertainty. Thirdly, the statistical power to detect weak correlations was limited by the sample sizes of the RPG sets. Therefore, these particular findings should be interpreted with caution and validated in larger future studies. Fourth, an important limitation is the lack of spatial resolution, as our analyses were derived from bulk tissue profiling. As a result, the spatial distribution of the identified transcriptomic signatures within the TME remains undefined. Employing spatial transcriptomics or single-cell sequencing in future studies will be essential to address this gap and substantiate our conclusions. Finally, although CT is widely used and relatively low-cost, combining other imaging modalities might enhance the robustness of the results. For example, Ryo et al. suggested a possible cutoff value 3.45 for maximum standardized uptake value on 18F-FDP PET/CT scans to identify high-grade IPA^49^. Therefore, future grading studies may require more extensive exploration.

In conclusion, our study identified radiopathomics phenotypes for IPA grading and investigated their biological significance, boosting the histopathological grading process and delivering more biological insights for IPA.

## Methods

### Patients

This multicenter retrospective study was conducted in accordance with the Declaration of Helsinki and was approved by the Medical Ethics Committee of The Affiliated Panyu Central Hospital, Guangzhou Medical University. Due to the retrospective design, patient informed consent was waived. All patient specimens underwent radical surgical resection and received a pathological confirmation of IPA. The patient data consisted of four datasets: (1) the Radiopathomics analysis set (approval No. PYRC-2021-006); (2) the Radiopathomics test set (approval No. RYRC-2024-275-01); (3) the TCGA validation set; and 4) the CPTAC validation set.

For the (1) Radiopathomics analysis set and (2) Radiopathomics test set, the two datasets were derived from patients consecutively enrolled at the local institution over different periods. The retrospective patient data in the Radiopathomics analysis set were from a previous study^31^ conducted by our team and were used for model development and testing. The Radiopathomics test set consisted of subsequently enrolled IPA patients and was used for independent model validation. The inclusion criteria were as follows: histologically confirmed IPA with curative surgical resection; complete data on clinical information, preoperative CT images, and HE-stained slides; and no history of other malignancies. The exclusion criteria were: (i) invasive mucinous adenocarcinoma and other adenocarcinoma variants (colloid adenocarcinoma, fetal adenocarcinoma, or enteric-type adenocarcinoma), (ii) severe artifacts in CT images that impair assessment, (iii) poor quality of slides affecting histological evaluation (e.g., inadequate staining, folds, extensive hemorrhage, necrosis, and blurring), (iv) insufficient tumor tissue, unless a definitive diagnosis could be made, and v) inadequate WSI scanning quality, such as extensive defocus and blurring. For the (3) TCGA validation set and (4) CPTAC validation set, the patients were derived from the TCGA-LUAD and CPTAC-LUAD datasets, respectively. They were used to validate the identified gene co-expression modules and hub genes related to grading. Patients with paired WSI and RNA sequencing data were retained. The exclusion criteria for WSI and CT images were the same as previously described.

Within the TCGA-LUAD dataset, clinical, RNA sequencing, and WSIs data are available on the Genomic Data Commons (GDC) Data Portal (https://portal.gdc.cancer.gov/). Radiological data about this dataset is stored on The Cancer Imaging Archive (TCIA) (www.cancerimagingarchive.net). Furthermore, in the CPTAC-LUAD dataset, patients’ clinical, CT, and WSIs can be retrieved from TCIA, while the corresponding RNA sequencing data can be accessed from the GDC Data Portal. It should be noted that the acquisition of all public datasets does not require patient consent, as the patient data has been appropriately anonymized.

### Histological grading

Without knowledge of patient clinical outcomes, two pathologists (reader 1, F.C. and reader 2, H.W.) with more than 15 years of experience in pulmonary pathology, reviewed all available H&E-stained tumor slides and downloaded WSI, grading the IPA according to the latest WHO classification^24^. According to the WHO classification, the histologic grade of IPA was categorized into three tiers: grade I, II, and III. For the purpose of this study, we stratified the patients into a High-grade group (grade III) and a Low-grade group (encompassing grades I and II). This stratification was based on their distinct prognostic outcomes and to address the issue of class imbalance in model development. Any disagreements between the pathologists were resolved through discussion. The grading criteria are as follows: grade I, lepidic predominant tumor; grade II, acinar or papillary predominant tumor, both with no or less than 20% of high-grade patterns; and grade III, any tumor with 20% or more of high-grade patterns (solid, micropapillary, or complex gland)^2^.

### Image acquisition and CT parameters

All patients at our institution underwent preoperative CT scans in the supine position while holding their breath. The CT images were retrieved from the Picture Archiving and Communication System (PACS). For local patients, CT imaging was performed using two scanners: the Discovery CT 750 HD (GE Healthcare, Chicago, IL, USA) and the Aquilion TSX-101A (Toshiba, Japan). The scan parameters for the Discovery CT 750 HD were as follows: scan type, helical; rotation time, 0.6 s; detector coverage, 40 mm; pitch, 1.375:1; tube voltage, 120 keV; tube current, automatic mA (50–350 mA) for a noise index of 13; scan field of view, Medium Body; scan slice thickness, 5 mm; reconstruction slice thickness and interval, 1.25 mm; reconstruction algorithms, ASIR-V level 40%; reconstruction kernel, standard and lung; matrix size, 512 × 512. For the Aquilion TSX-101A, the scan parameters were as follows: tube voltage, 120 kV; tube current, 250 mA; pitch, 0.844; collimation, 1.6 mm; matrix size, 512 × 512; pixel spacing, 0.8 mm; reconstruction kernel, FPB; reconstructed slice thickness, 1 mm.

### VOIs annotation

For all patients, reader 3 (Z.A. with more than 10 years of work experience in CT diagnosis of lung adenocarcinoma) imported the CT images into ITK-Snap software (version 3.8.0; www.itksnap.org) and annotated the 3D volumes of interest (VOIs) in the transverse plane without knowledge of the pathological outcomes. Two months later, reader 3 and reader 4 (Q.J.H. with more than 10 years of work experience) annotated the VOIs for the CT images of 40 randomly selected patients from the radiopathomics analysis set to assess intra- and inter-observer reproducibility.

Reader 5 with four years of experience in lung adenocarcinoma research selected the most representative slide for each patient (sufficient for diagnosing the grade of IPA). The acquisition of WSI images for public datasets was as previously described, with obtained WSI images in SVS format at 20× or 40× magnification. Finally, pathologist reader1 and reader2 imported all WSI images into Qupath software (Version: 0.3.2, https://qupath.github.io/) for tumor preview and annotation of the three high-grade patterns (solid, micropapillary, or complex gland) and non-high-grade patterns in IPA.

### Image preprocessing and feature extraction

We performed corresponding preprocessing on CT and WSI images and designed feature extraction methods. Given the large size of WSIs, all WSIs were divided into 1024 × 1024 pixel tiles, with patches containing predominantly background or non-tumor tissue (less than 50% tumor tissue) excluded. Subsequently, all patches underwent color normalization using the methodology developed by Vahadane et al.^50^. Feature extraction from WSI images was completed in two steps: feature extraction at the patch level and aggregation to the WSI level. The training of the DL model was implemented on the OnekeyAI (3.0.3) platform and was conducted using an NVIDIA RTX A3000 GPU.

For the HRF: We designed and extracted 1316 features using the Artificial Intelligence Kit (AK) software (version 3.0.0.R, GE Healthcare). Additional details about HRF are available on the PyRadiomics website (https://pyradiomics.readthedocs.io/en/latest/). First, we preprocessed the CT images by resampling the voxel sizes (three-dimensional pixels) of all images to 0.5 × 0.5 × 0.5 mm using the nearest-neighbor interpolation method to standardize the resolution. Subsequently, we set the image transform types and parameters: LoG (Laplacian of Gaussian) with Sigma values of 2.0 and 3.0. Wavelet transformation at Level 1 is applied for multi-scale analysis to extract features across different frequency components. LBP(3D) (Local Binary Patterns) with Level 2, Radius 1, and Subdivision 1 captures local texture patterns, enhancing texture features. Additionally, the BinWidth is set to 25, which determines the bin width for gray level histograms. Finally, the extracted feature types and quantities are as follows: FirstOrder includes 252 features, comprising gray level statistical measures such as mean, median, and standard deviation. Shape(3D) consists of 14 features that describe the geometric shape of the ROI, including volume and surface area. GLRLM (Gray Level Run Length Matrix) provides 224 features, assessing texture by describing the spatial distribution of gray level runs. GLSZM (Gray Level Size Zone Matrix) also contributes 224 features, evaluating the size and distribution of homogeneous gray level zones in the image. GLCM (Gray Level Co-occurrence Matrix) offers 336 features that provide texture information by describing contrast, correlation, energy, and other gray level dependencies. GLDM (Gray Level Dependence Matrix) includes 196 features, describing the dependency of gray levels to assess contrast and complexity. Lastly, NGTDM (Neighboring Gray Tone Difference Matrix) contains 70 features, evaluating the spatial dependence of gray tones and capturing fine texture details.

For the DLRF: Before feature extraction, we selected the slice with the largest ROI area on the cross-sectional CT images of each patient and cropped a rectangle that fully contained the ROI for each patient. Next, we applied a window level (WL) of −700 and a window width (WW) of 1200 to transform the grayscale images, standardizing the background information and minimizing noise interference. We then used Resnet18, pre-trained on the ImageNet dataset (http://www.image-net.org), as the backbone, and fine-tuned the model on the radiopathomics training set for the grade task. The hyperparameters for the grade classification task were set as follows: initial learning rate, 0.01; number of epochs, 100; batch size, 32. Lastly, the second-to-last layer of the fine-tuned model was used as the feature extraction layer.

For the HPF: We randomly selected 50 patches from each patient (including all tiles if there were fewer than 50) and used CellProfiler software (version 4.2.1, https://cellprofiler.org/) to construct a pipeline for segmentation and extraction of the handcrafted pathomics features. In the process of extracting histological image features using CellProfiler, we first used the “UnmixColors” module to separate each dye in histological stained images, generating corresponding grayscale images. Next, we utilized the “IdentifyPrimaryObjects” module to identify primary objects (nuclei) in the image, recording the object count and centroid coordinates. Then, the “IdentifySecondaryObjects” module was used to identify secondary objects (cells) based on the identified primary objects, recording the secondary object count and centroid coordinates. Following this, the “MeasureObjectSizeShape” module measured various area and shape features of the identified objects, including area, volume, perimeter, form factor, eccentricity, and primary axis length. Subsequently, the “MeasureTexture” module measured texture features within images and objects, such as angular second moment, contrast, correlation, variance, and entropy. Afterward, the “MeasureObjectIntensity” module measured the intensity features of objects, such as integrated intensity, mean intensity, standard deviation of intensity, maximum intensity, and minimum intensity. Finally, the “MeasureObjectIntensityDistribution” module measured the spatial distribution of intensities within each object, including the fraction of total stain at a given radius, mean fractional intensity, and the coefficient of variation of intensity within a ring. The average of the features from all selected tiles in each patient was taken as the handcrafted pathomics features for that patient. Previous studies have reported the role of tumor nuclei and cytoplasm in tumor prognosis and grading^38,51^.

For the DLPF: we employed a MIL approach. In the MIL framework, feature extraction was divided into two main steps: (i) First, we initialized the Resnet18 network parameters using weights pre-trained on the ImageNet dataset. Subsequently, we fine-tuned the model using patches from the radiopathomics training set, which included both high-grade and non-high-grade growth patterns. The fine-tuned model was then used to predict the probability of patches from all patients. The model’s hyperparameters were as follows: batch size was set to 32, the learning rate was set to 1e-2, and the learning rate decayed by half every 30 epochs. The optimizer used for training was stochastic gradient descent. (ii) After obtaining the patch-level probabilities for each patient, we aggregated the probabilities of all patches to derive WSI-level features. In our study, we referenced the method of Cao et al.^40^, using the Bag-of-Words (BoW) approach, where each patch was mapped to a TF-IDF floating-point variable, and a TF-IDF feature vector was computed to represent the WSI. These features were then used to train traditional machine learning classifiers for IPA grading. Notably, to assess the importance of distinct image regions in the predictions generated by the DL model, we employed the Gradient-weighted Class Activation Mapping (Grad-CAM) technique for visualization^52^. Grad-CAM utilizes gradient information to attribute significance values to the feature map, illuminating the areas where the model directs its attention during prediction.

### Feature selection and model construction

Feature selection and model construction were based on radiopathomics training set data. Before analysis, all features were normalized using Z-Score to reduce biases from different dimensions. Different modality features were screened through the following steps: First, ANOVA was used to remove features that did not significantly affect the results; second, Spearman correlation analysis was performed to remove redundant features, where features with correlation coefficients ≥0.8 or ≤−0.8 were regarded highly correlated, and only one of them was retained; subsequently, the Boruta algorithm (a wrapper algorithm based on random forests) was used to iteratively evaluate the importance of each feature and eliminate features with importance significantly lower than that of shadow features^53^. Finally, the LASSO analysis was employed to reduce the number of features further and select those with coefficient weights greater than zero as the most important subset of features^54^. Furthermore, we quantified the biological signal strength of each feature relative to inter-institutional variability (noise) using the SNR^55,56^. Features with SNR ≥2 were deemed highly reliable^56^. This assessment aimed to evaluate the stability of model-derived features across institutions. After feature selection, we trained 11 machine learning classification algorithms, including Logistic Regression (LR), Naive Bayes, K-Nearest Neighbors, Random Forest (RF), Support Vector Machine, Extra Trees, XGBoost, LightGBM, Gradient Boosting, AdaBoost, and Multilayer Perceptron (MLP), on each data modality. The models were then tested using a hold-out set, and the best-performing model was chosen for subsequent analysis. With the MLP demonstrating superior performance among all machine learning models and thus being selected as the optimal model. To evaluate the performance of models integrating macro-radiological and micro-pathological features, our study implemented ensemble learning, combining features from different modalities. We included all selected features in the training of the 11 algorithms and integrated the results of all models using a “weighted voting” method to create the DHRPs model.

### Model performance and clinical utility

Evaluation of each model included the assessment of performance and clinical utility using measures such as the area under the receiver operating characteristic curve (AUC), accuracy, sensitivity, specificity, negative predictive value, positive predictive value, calibration curves, and clinical decision curve analysis. Additionally, DeLong’s test was employed to compare the AUCs of different models.

### Model vs. pathologist performance comparison and consistency analysis

Two board-certified pathologists specializing in lung adenocarcinoma subtyping (LSL and CQX, with 2 and 3 years of specific experience, respectively) performed a blinded evaluation of WSIs from 102 patients in the Radiopathomics test set, without prior knowledge of the original pathological diagnoses. The performance of the DHRPs model was compared against that of the pathologists based on ROC analysis, sensitivity, and specificity. To ensure a fair comparison, an operating-point matching strategy was adopted to mitigate biases arising from differences in decision thresholds: for each pathologist’s sensitivity (or specificity) level, the model’s decision threshold was adjusted accordingly to match that level, enabling a direct comparison of specificity (or sensitivity) under equivalent sensitivity (or specificity) conditions. Statistical significance was assessed using McNemar’s test. Additionally, inter-observer agreement between the two pathologists regarding IASLC grading and dominant subtype classification was further evaluated using Cohen’s kappa analysis. The coefficient values were interpreted as follows: 0–0.20 (“poor”), 0.21–0.40 (“fair”), 0.41–0.60 (“moderate”), 0.61–0.80 (“substantial”), and 0.81–1.00 (“almost perfect”).

### RNA sequencing

Paired-end sequencing was performed using the Illumina HiSeq high-throughput sequencing platform. During the quality control stage, only those samples with an RNA integrity number (RIN) ≥ 5.0 were included in this study. The RNA-seq reads were aligned to the reference human genome (hg19). RNA sequencing data was converted from fragments per kilobase transcriptome per million reads (FPKM) into transcripts per thousand base million (TPM).

### Gene co-expression module identification

To explore the potential biological processes underlying high- and non-high-grade IPA groups, we conducted WGCNA on the RPG training set^57^. This analysis clusters numerous genes into several modules, with genes within the same module typically reflecting similar biological functions. Prior to conducting WGCNA, we preprocessed the gene expression data. Initially, we mitigated batch effects between different datasets using the ComBat method and applied log transformation to minimize data heterogeneity. Following that, we narrowed down our focus to the top 5000 genes with the highest median absolute deviation (MAD) to streamline computational complexity and eliminate background noise stemming from genes with low variability. Subsequent to this, using the RPG training set, we harnessed the R package “WGCNA” to conduct WGCNA on the selected genes. Commencing with the clustering of samples to identify and remove outliers, we proceeded to utilize network topology analysis to pinpoint the optimal soft threshold (RsquaredCut, 0.85). After that, employing the optimally determined soft threshold, we built a scale-free network to cluster highly interconnected genes into several functionally akin gene co-expression modules (minModuleSize, 30; mergeCutHeight, 0.35). Each module is represented by Module Eigengene (ME).

To select highly reproducible modules, we calculated Zsummary statistics between the RPG training set and TCGA validation set and between the RPG training set and CPTAC validation set. Modules with Zsummary ≥10 were retained, as these modules are considered highly reproducible^58^. Subsequently, the first principal component of the highly reproducible modules (representing the genes within each module) was correlated with phenotypes using Spearman correlation analysis (*p* < 0.05 indicating significant correlation), identifying gene modules significantly associated with grade. The reproducibility of the correlation between module and grade will be evaluated in the TCGA and CPTAC validation sets. To evaluate the robustness of the identified associations between the grading phenotype and the modules, a post-hoc statistical power analysis was performed given our sample size.

### The biological basis of IPA grade, hub genes, and interpretation of radiopathomics features

We conducted the following analyses on the RPG training set to explore the biological basis at different grades, the hub genes related to grading, and the driving pathways behind radiopathomics features: (1) First, we conducted pathway enrichment analysis for genes within grade-related modules. Using the ClusterProfiler package, we queried the following seven annotated databases for each grade-related module to understand the biological functions of each module, with false discovery rate (FDR) < 0.05, indicating significant enrichment: Kyoto Encyclopedia of Genes and Genomes (KEGG), Gene Ontology (GO), Hallmark, Reactome, BioCarta, Pathway Interaction Database (PID), and WikiPathways (WP). For each enriched pathway, we calculated its GSVA score to assess the activity level of the predefined gene sets in different patients. (2) Second, to identify differences in biological pathways between high- and non-high-grade IPA, we performed a Mann-Whitney U test on the GSVA scores of each pathway (FDR < 0.05 indicating significance). To further investigate the biological basis of different grade groups, we also conducted GSEA within the modules to determine if the predefined gene sets (Seven gene sets from the MSigDB database are the same as described above.) showed significant and consistent differences between the high- and non-high-grade IPA. (3) Third, we identified hub genes connected to grading within each module. Genes with |MM| > 0.80 and GS > 0.20 were selected as hub genes. (4) Finally, we utilized Pearson correlation analysis to analyze the link between the GSVE scores of significantly enriched pathways and radiopathomics features (FDR < 0.1 indicating significant correlation), and the correlated pathways were used to annotate radiopathomics features. All analyses were validated using the RPG-TCGA and RPG-CPTAC external validation sets. We assessed the detection limit and statistical power of our correlation analyses by calculating both the MDCC (*α* = 0.05, power = 0.8) and the achieved power for each observed feature-pathway correlation (two-tailed, *α* = 0.05).

### Statistical analyses

All analyses were conducted using R (version 4.1.1, https://www.R-project.org) and Python (version 3.9.6, https://www.python.org). Continuous variables were described as mean ± standard deviation, and categorical variables were described as frequencies and percentages. The Mann-Whitney U test was employed for continuous variables, while Fisher’s exact or chi-square test was utilized for nominal variables. The Kruskal-Wallis test was used for comparisons involving multiple groups. The achieved statistical power for the observed correlations was calculated post-hoc using the pwr.r.test function in R, with a significance threshold of *α* = 0.05. The ICCs were used to assess the agreement of features. The criterion for statistical significance was set as a two-tailed *P* < 0.05. For multiple testing, the P-values were also adjusted using the FDR method. Some illustrations were generated using BioRender.com.

## Supplementary information

Supplementary Information

## Data Availability

The clinical data, WSI, and CT images from the Radiopathomics analysis set and Radiopathomics test set used in this study (approved by the IRB) are not publicly available to ensure the protection of patient privacy. However, access may be granted upon reasonable request to the corresponding author, Z.X. ([xiangzhiming@pyhospital.com.cn](mailto:xiangzhiming@pyhospital.com.cn)). Any data shared will be restricted to non-commercial use, with all sensitive patient information thoroughly removed. Typically, requests for data access are processed within two weeks. Public datasets (TCGA-LUAD and CPTAC-LUAD) can be accessed through the following links: https://portal.gdc.cancer.gov/, https://www.cancerimagingarchive.net/collection/tcga-luad/, and https://www.cancerimagingarchive.net/collection/cptac-luad/.

## Code availability

The code supporting this study is available at https://github.com/holsenyang/Radio-Patho-Genomics.
