# Supplementary material for: Bio-interpretable ensemble learning model for invasive pulmonary adenocarcinoma grade using CT and histopathology images
Source: NPJ Precis Oncol. 2025 Dec 18;10:41. doi: 10.1038/s41698-025-01239-3 (PMC12824164; doi:10.1038/s41698-025-01239-3)
Supplement: Supplementary file 1 — Supplementary Information [file 41698_2025_1239_MOESM1_ESM.pdf]

Supplementary Information

Bio-interpretable ensemble learning model for invasive pulmonary adenocarcinoma grade using CT and histopathology images

Zhihe Yang [1 2], Fan Li [3], Qijia Han [4], Zhu Ai [4], Minyi Wu [4], Qiuxing Chen [4], Siqi Qu [4], Lingxiang Liu [2], Haowen Yan [1 2], Guorong Zou [1 2], Fang Chen [5], Hao Wang [5], Zhiming Xiang [4]

[1] Cancer Institute of Panyu District, The Affiliated Panyu Central Hospital, Guangzhou Medical University, Guangzhou, P. R. China

[2] Department of Oncology, The Affiliated Panyu Central Hospital, Guangzhou Medical University, Guangzhou, P. R. China

[3] Department of Laboratory Medicine, Guangdong Provincial People's Hospital Ganzhou Hospital, Ganzhou Municipal Hospital, Ganzhou, P. R. China

[4] Department of Radiology, The Affiliated Panyu Central Hospital, Guangzhou Medical University, Guangzhou, P. R. China

[5] Department of Pathology, The Affiliated Panyu Central Hospital, Guangzhou Medical University, Guangzhou, P. R. China

Corresponding author: Zhiming Xiang, Email: xiangzhiming@pyhospital.com.cn

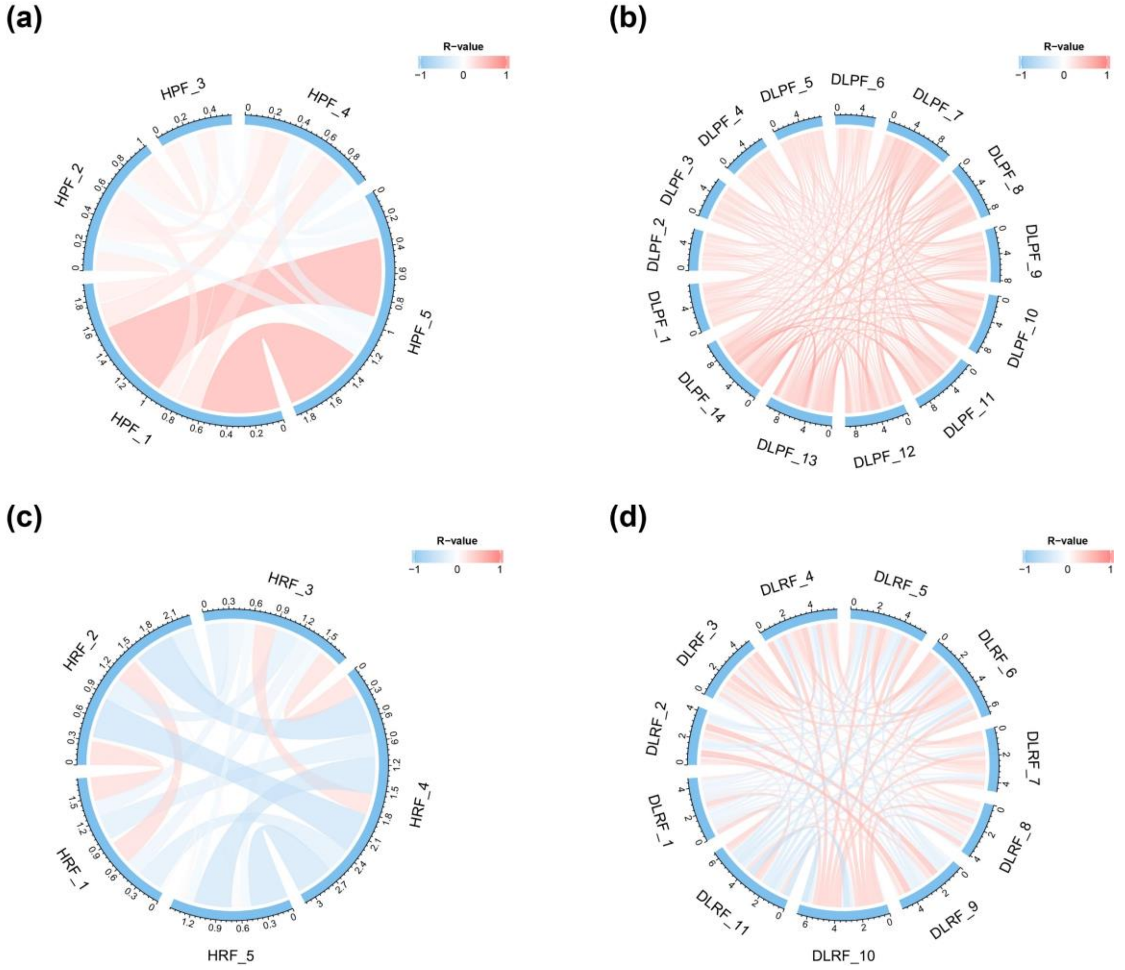

Supplementary Figure S1. The Spearman correlation (R) among each feature across different feature categories.

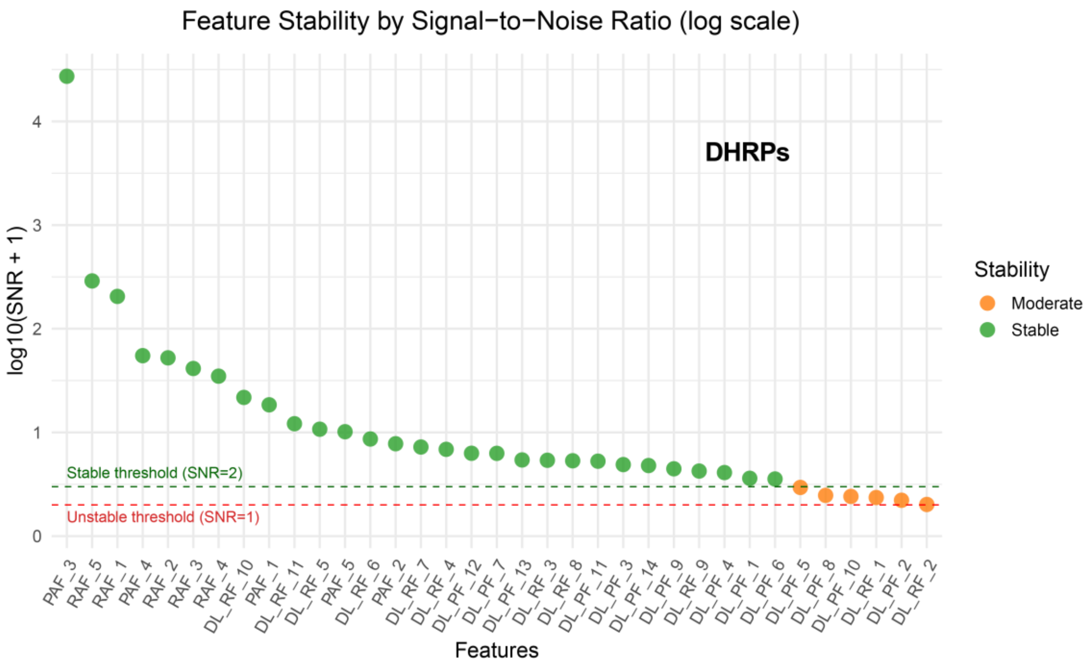

Supplementary Figure S2. Signal-to-noise ratio (SNR) of all features in the DHRPs model.

All features utilized in our final integrated DHRPs model exhibited an SNR greater than 1 (accepted threshold indicated by the dashed line). Handcrafted features demonstrated superior robustness with an SNR ≥ 2, while a subset of DL features, despite having SNRs between 1 and 2, remained within the acceptable range of stability.

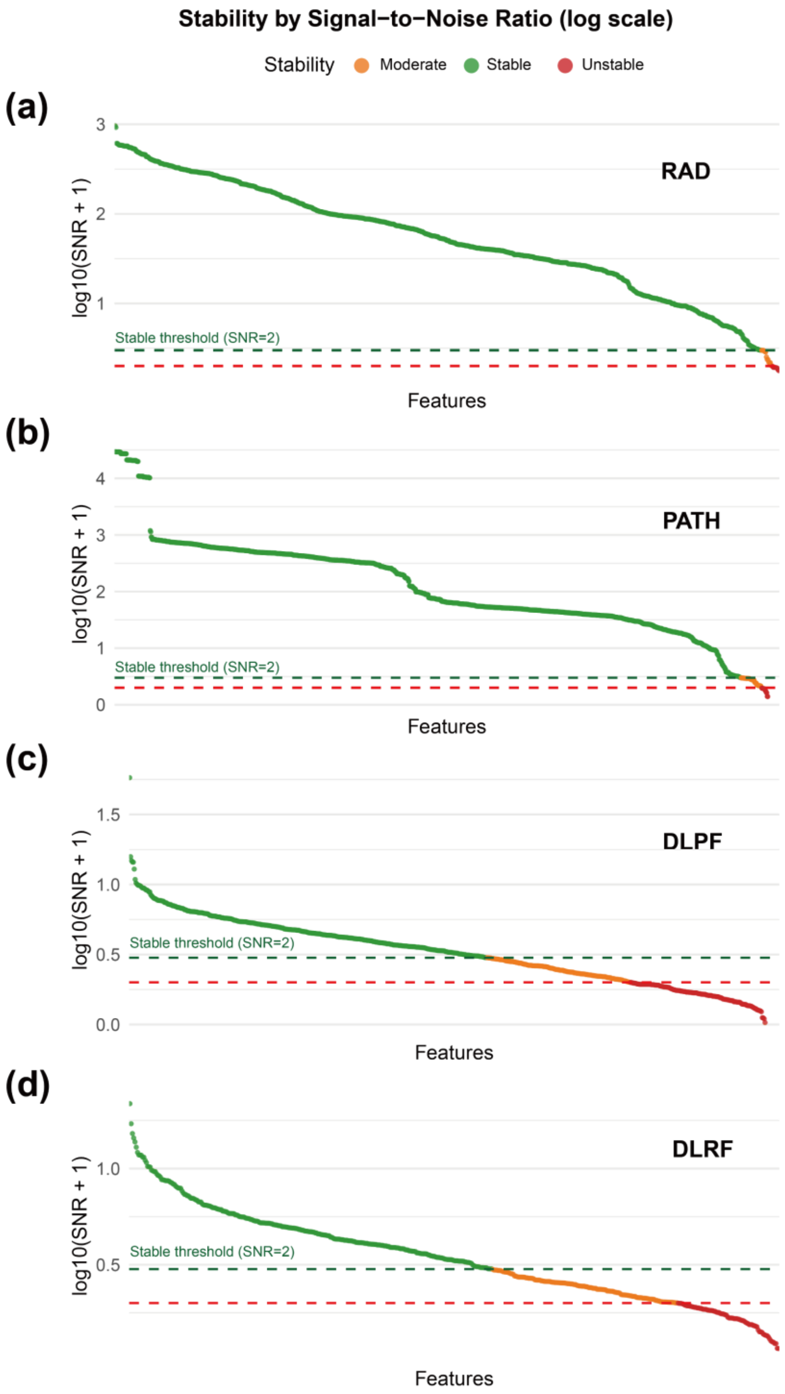

Supplementary Figure S3. Signal-to-noise ratio distribution of four feature categories.

This figure displays the distribution of SNR values for each of the four feature categories analyzed. The distributions show that most handcrafted radiomics (HRF) and pathomics (HPF) features possess SNR values above the robustness threshold (**a** and **b**). DL features, however, showed greater variability and a higher prevalence of low-SNR features initially, suggesting a susceptibility to inter-center distribution shifts (**c** and **d**).

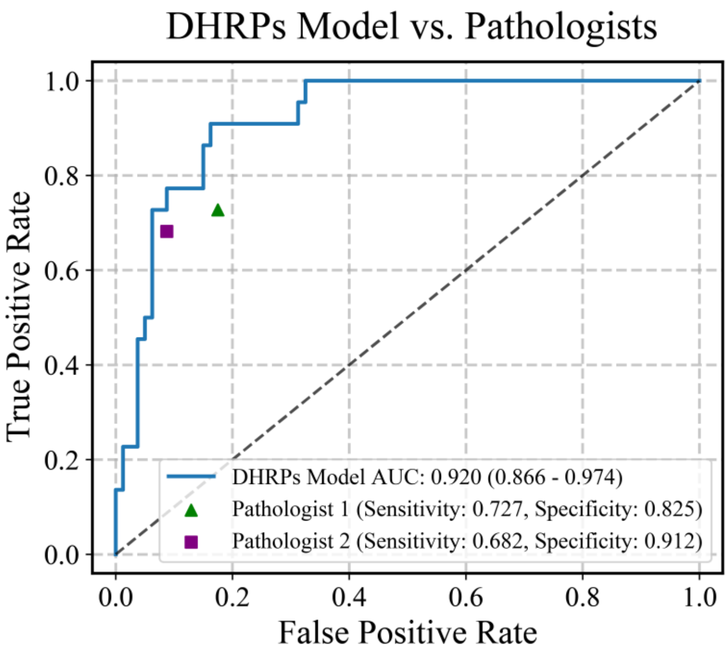

Supplementary Figure S4. Performance comparison between the DHRPs model and pathologists.

The diagnostic performance of the proposed DHRPs model is directly compared against that of two practicing pathologists. The operating points of both pathologists fall below and to the right of the model's ROC curve.

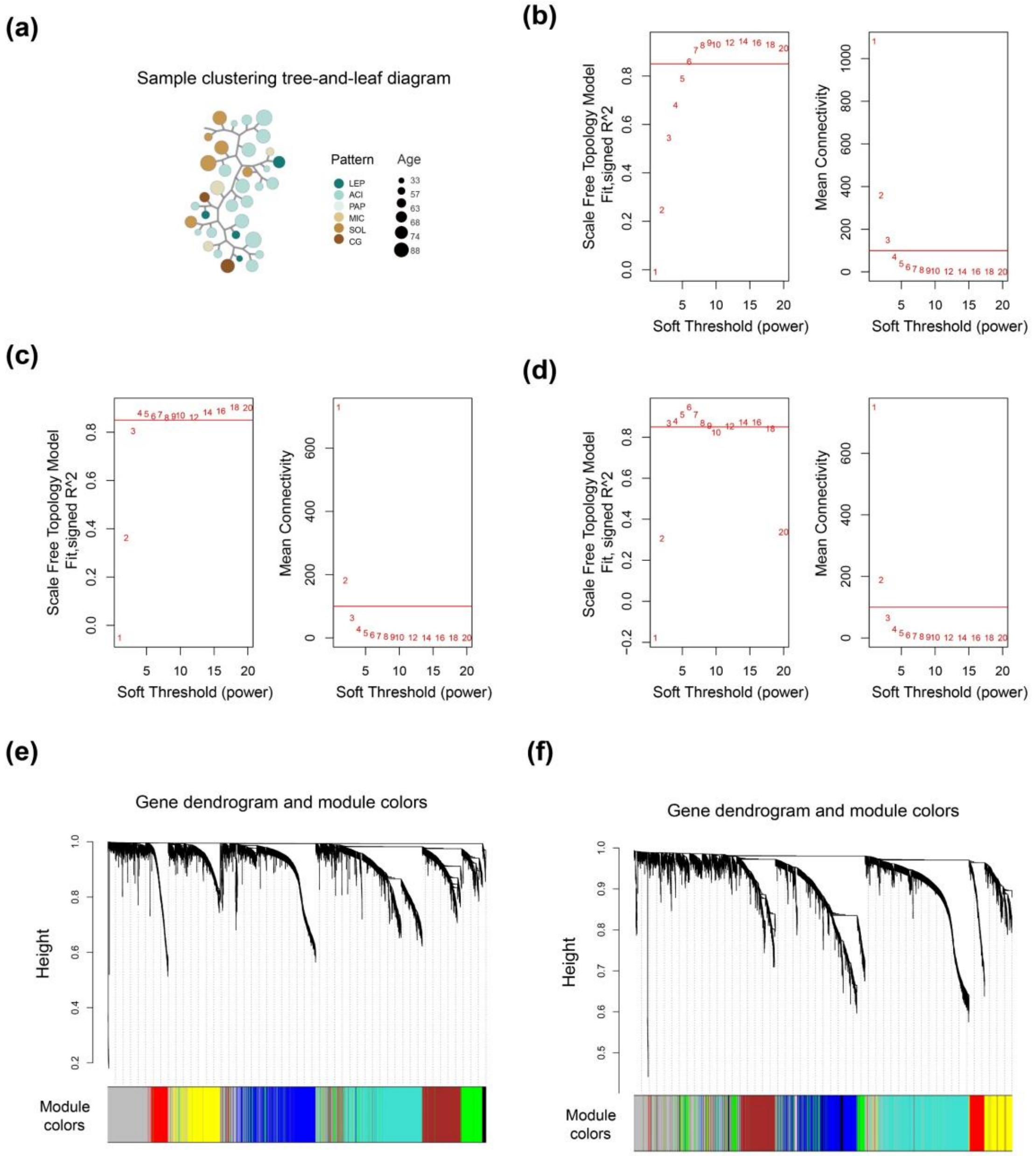

Supplementary Figure S5. Gene co-expression network analysis results.

(**a**) Clustering tree-and-leaf diagram of samples in the PRG training set. Determination of soft-thresholding power value in WGCNA based on the RPG training set (**b**), TCGA validation set (**c**), and CPTAC validation set (**d**). Clustering dendrogram of gene co-expression modules in TCGA validation set (**e**), and CPTAC validation set (**f**). WGCNA, weighted gene co-expression network analysis; TCGA, The Canter Genome Atlas; CPTAC, Clinical Proteomic Tumor Analysis Consortium.

(a)

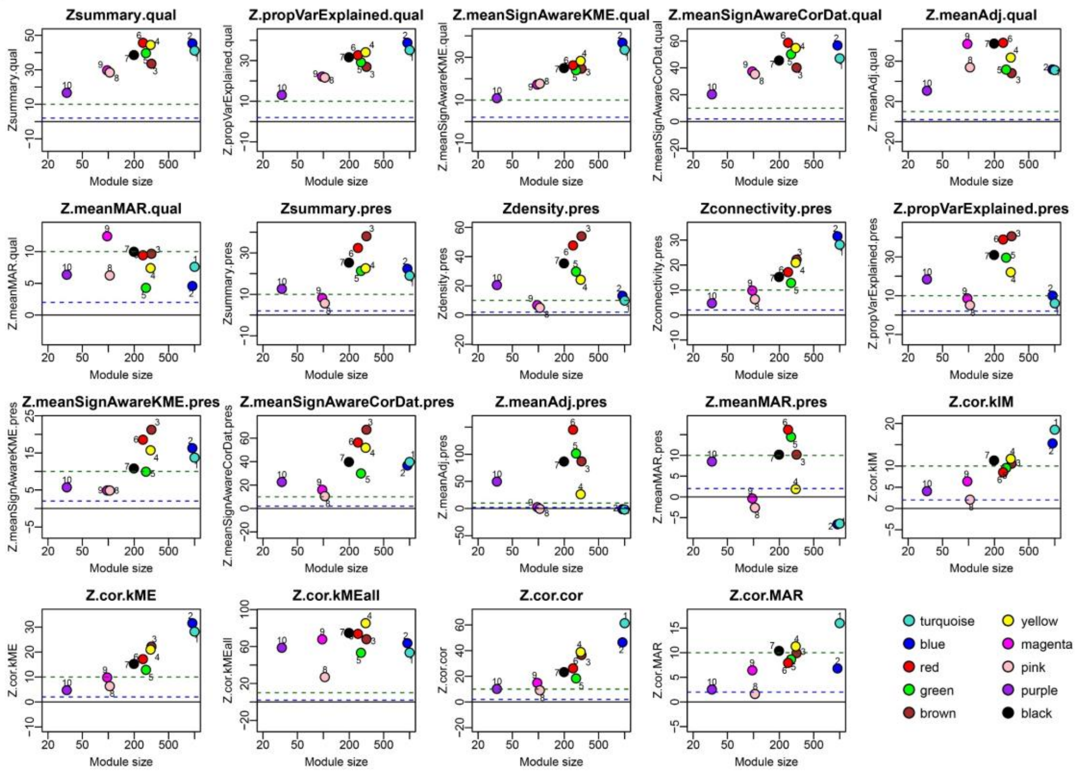

(b)

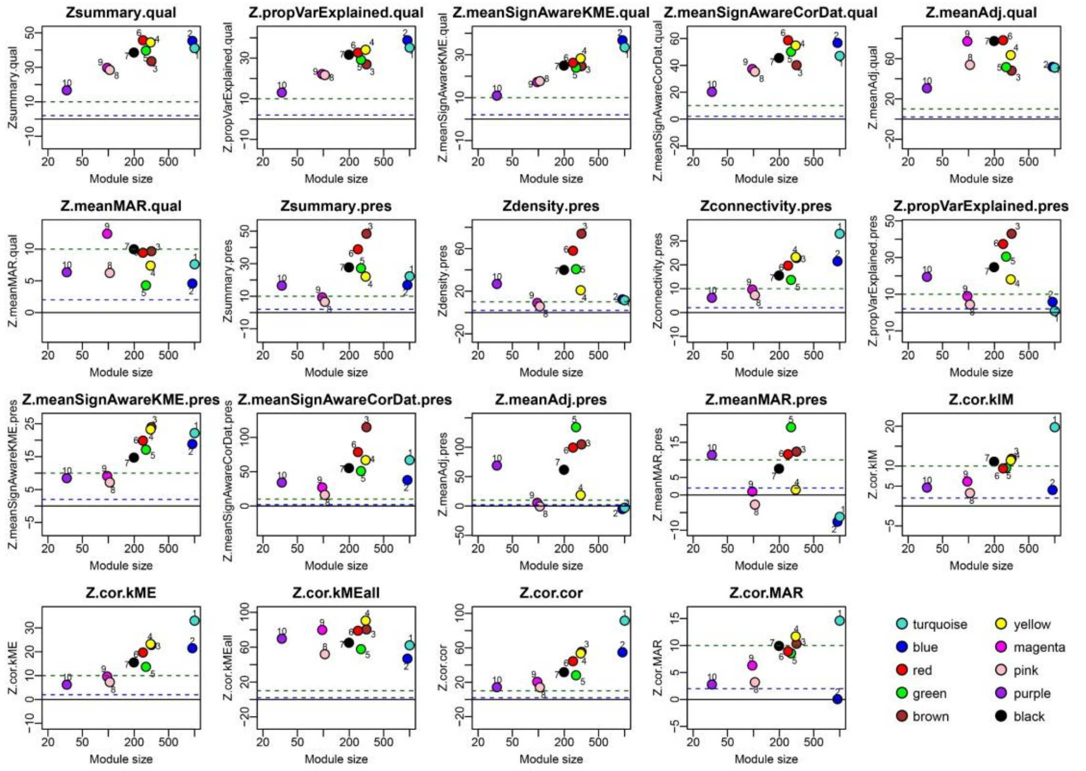

Supplementary Figure S6. Density and connectivity statistics.

The figure shows the quality of modules constructed in the RPG training set that are retained in TCGA validation set (**a**) and CPTAC validation set (**b**). Different colors represent different modules. The horizontal axis represents

module size (number of genes), while the vertical axis displays Z statistics for different types of analysis. The blue and green dashed lines represent $Z = 2$ and $Z = 10$, respectively. $Z < 2$ indicates non-conservation, $2 < Z < 10$ indicates mild to moderate conservation, and $Z > 10$ indicates high conservation.

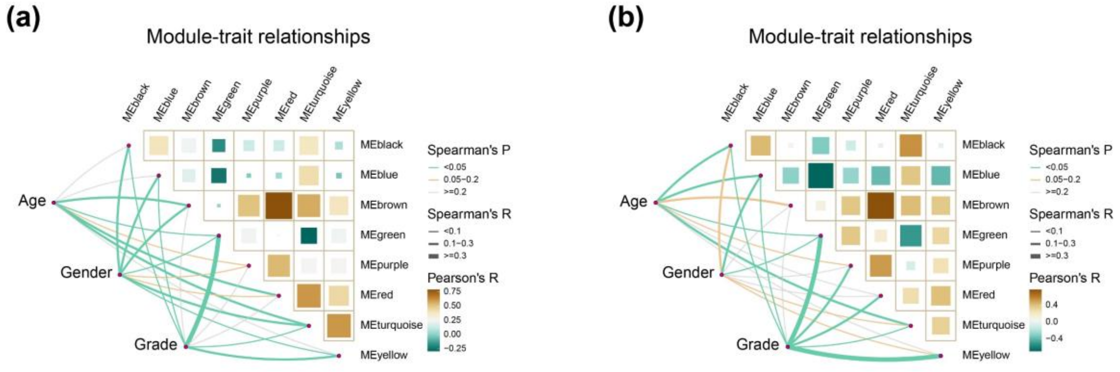

Supplementary Figure S7. Correlation among modules and the association between modules and phenotypes.

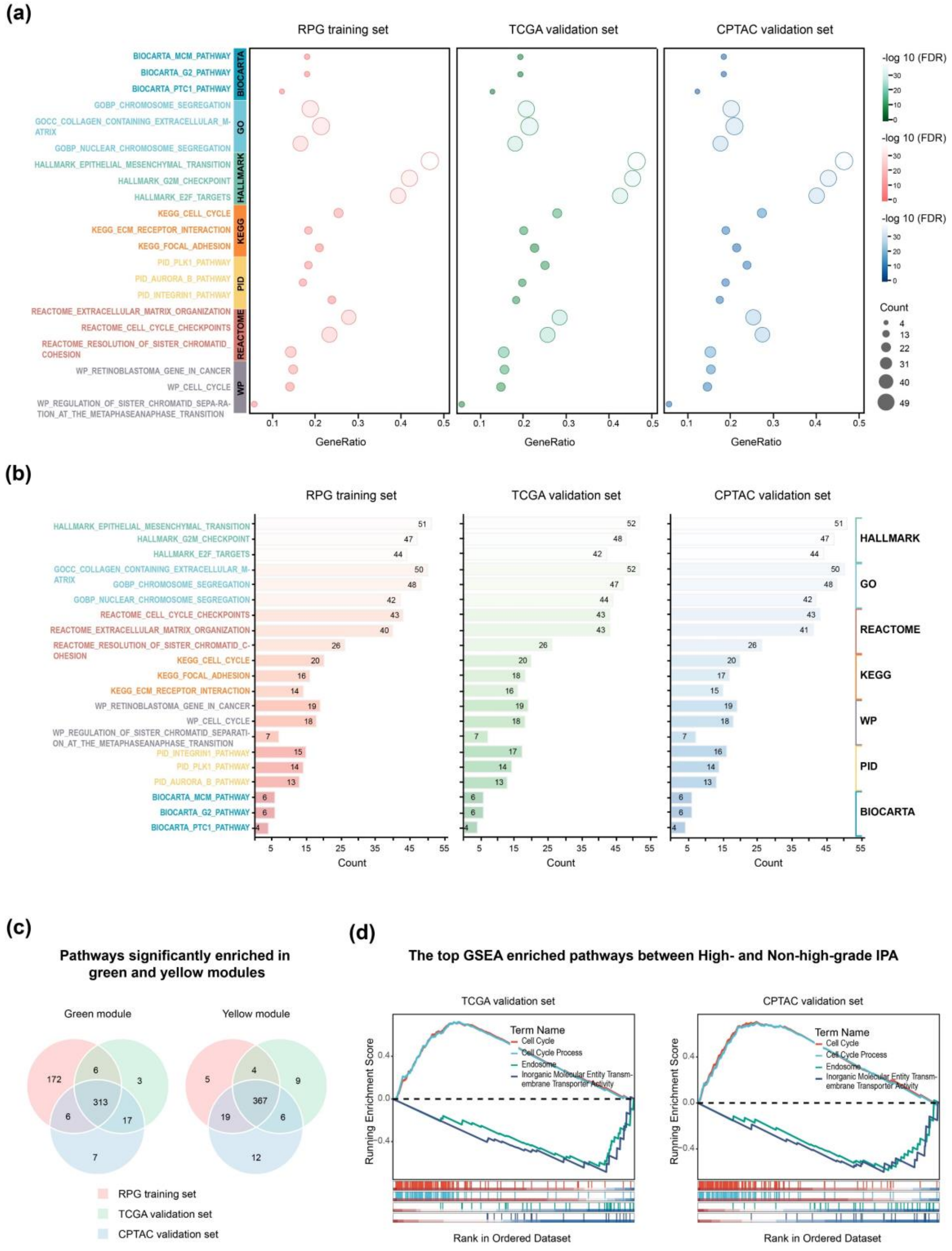

Supplementary Figure S8. Biological processes related to IPA grading.

(**a**, **b**), Bubble plots and bar charts displaying the top three significant pathways within each of the seven gene sets—Kyoto Encyclopedia of the Genome (KEGG), Gene Ontology (GO), Hallmark, Reactome, BioCarta, Pathway Interaction Database (PID), and WikiPathways—after pathway enrichment analysis of genes within the green and yellow modules in the RPG training set (left), TCGA validation set (middle), and CPTAC validation set (right). (**c**) Venn diagram of significantly enriched overlapping pathways within the green (left) and yellow (right)

modules across the three datasets. Most pathways are reproducible within the green and yellow modules. (**d**) Gene set enrichment analysis results. The figure shows the two most significantly upregulated and two most significantly downregulated pathways in the TCGA validation set (left) and the gene set enrichment analysis results for these four pathways in the CPTAC validation set (right).

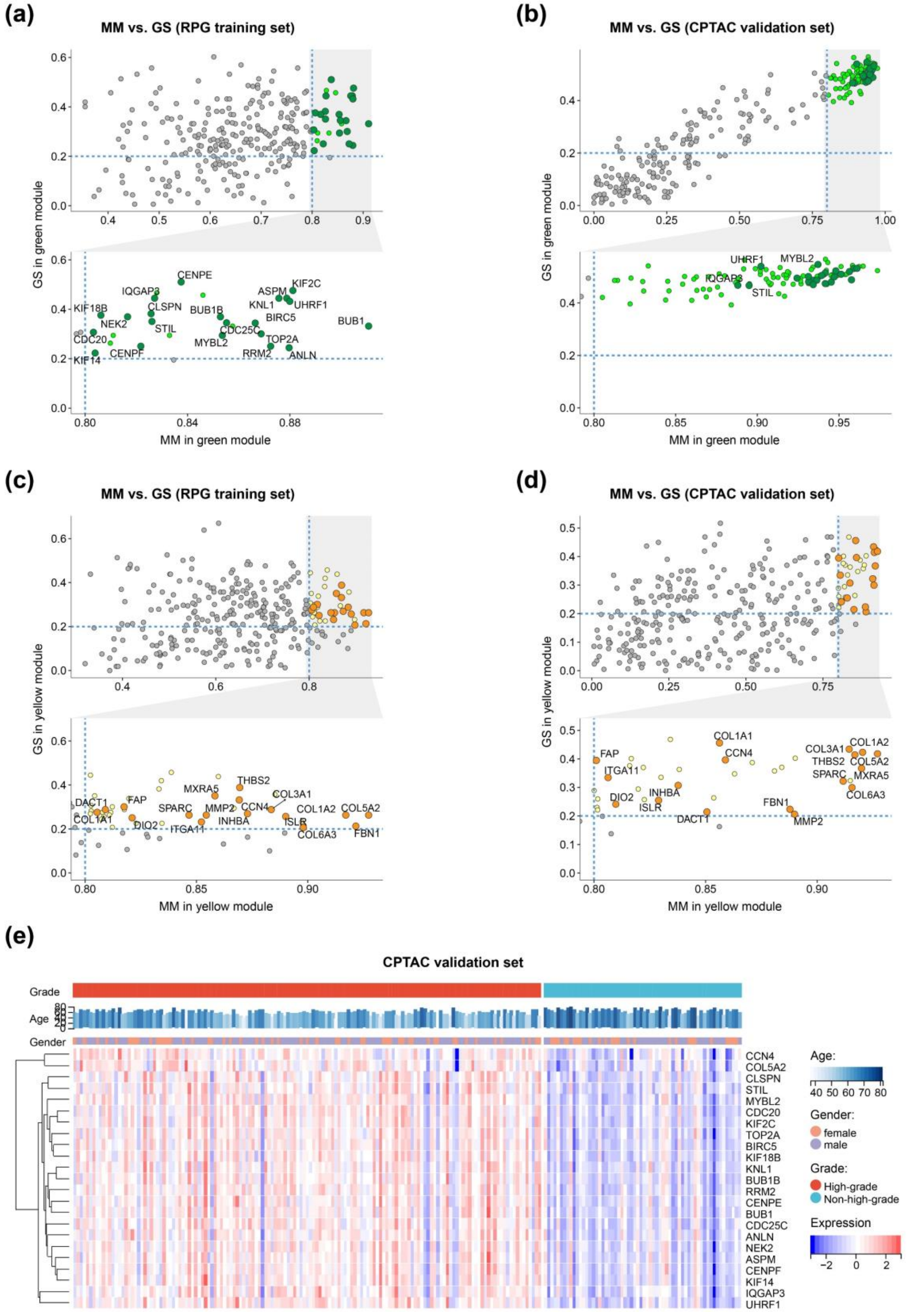

Supplementary Figure S9. Identification and visualization of grading-related hub genes.

Scatter plots of MM (representing gene-module correlation) and GS (representing gene-trait correlation) within the green module (**a**, **b**) and the yellow module (**c**, **d**) in the RPG training set and CPTAC validation set. The

horizontal blue dashed line indicates GS = 0.2, while the vertical blue dashed line indicates MM = 0.8. Each scatter point represents a gene within the module. Genes with |MM| > 0.80 and GS > 0.20 are considered important elements within the module. Dark green and orange colors indicate reproducible hub genes within the green and yellow modules, respectively. **(e)** Heatmap of Z-score standardized expression of the 23 identified reproducible hub genes in the CPTAC validation set. GS: gene significance; MM: module membership.

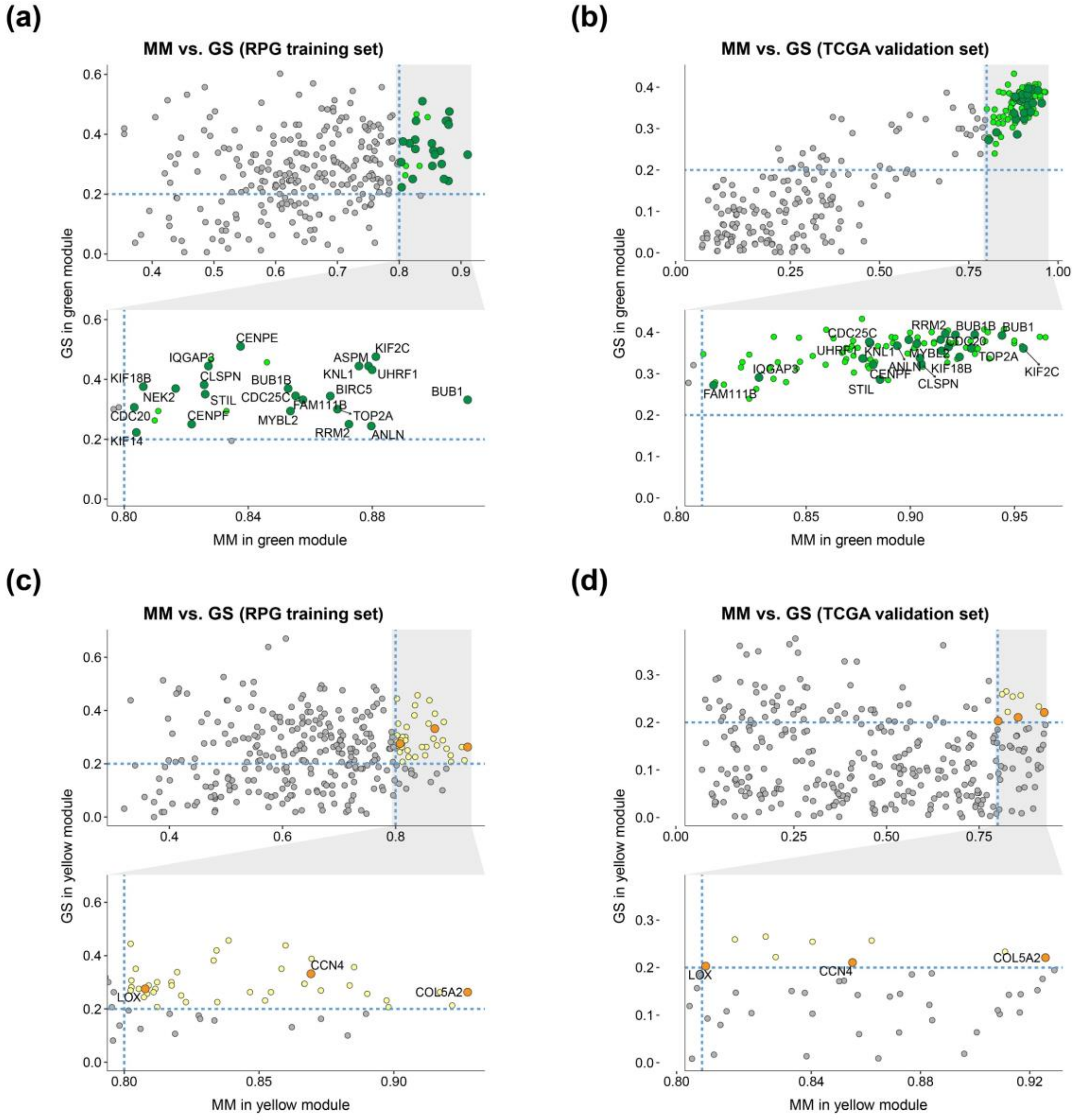

Supplementary Figure S10. Identification of grading-related hub genes.

Scatter plots of MM (representing gene-module correlation) and GS (representing gene-trait correlation) within the green module (**a**, **b**) and the yellow module (**c**, **d**) in the RPG training set and TCGA validation set.

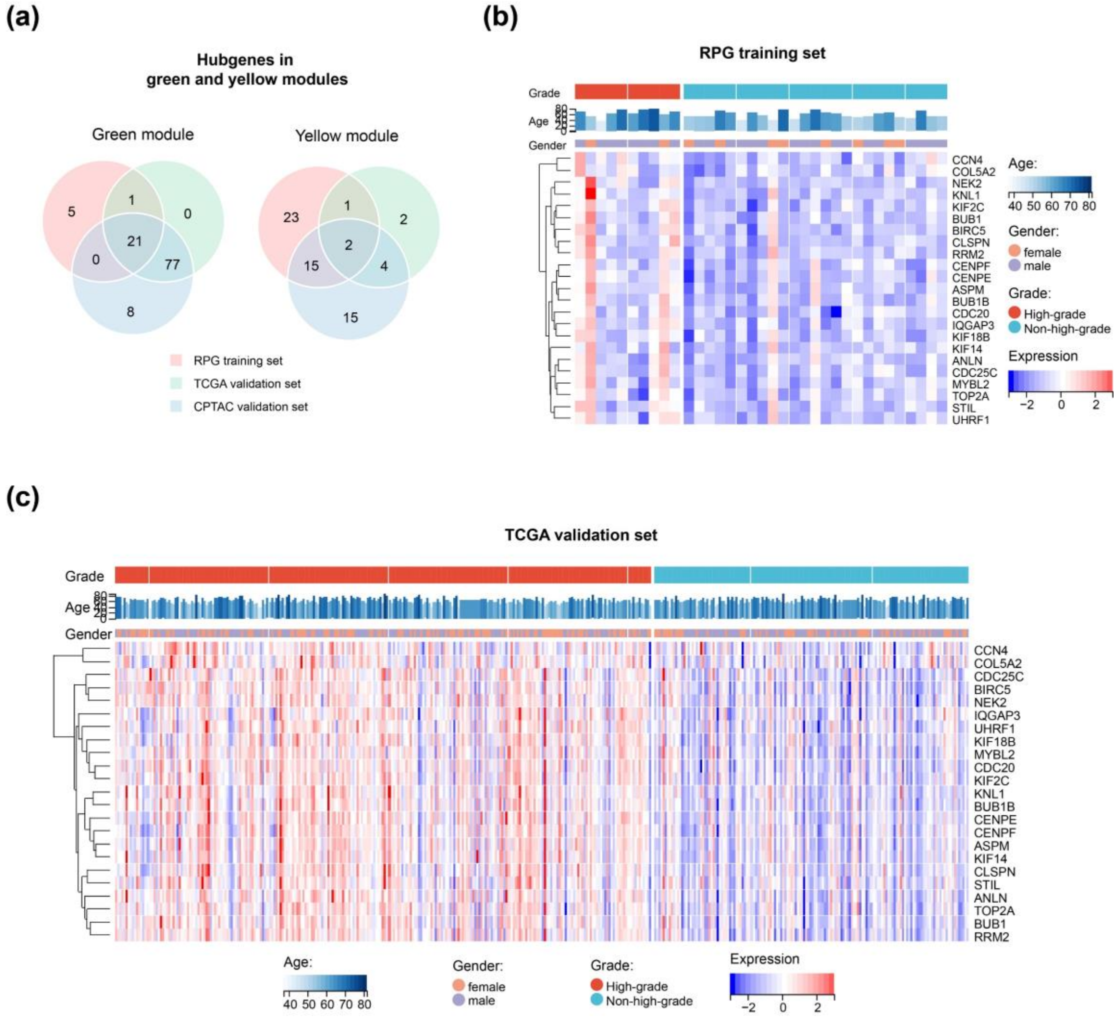

Supplementary Figure S11. Visualization of grading-related hub genes.

(a) Venn diagram of hub genes within the green (left) and yellow (right) modules across the three datasets. Heatmap of Z-score standardized expression of the 23 identified reproducible hub genes in the RPG training set (**b**) and TCGA validation set (**c**).

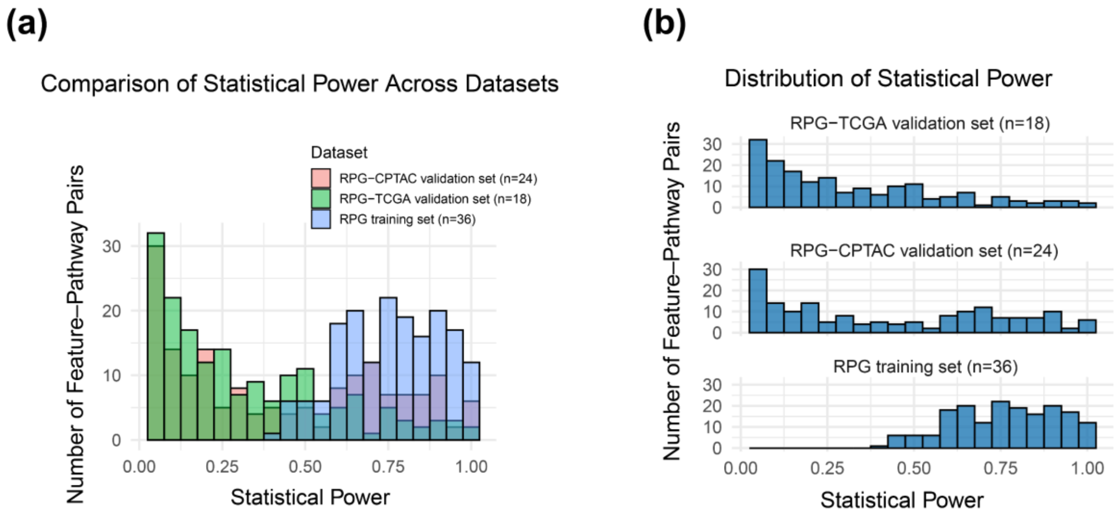

Supplementary Figure S12. Power analysis of feature-pathway correlations in multi-center datasets.

(a) Power of significant associations per cohort. The detection of medium-to-high power (≥ 0.6) associations in small datasets confirms the stability and substantial effect size of the identified robust signals. (b) Sample size effect on statistical power. The increasing proportion of high-power associations with larger sample sizes (n=18 -> n=36) aligns with statistical theory, underscoring the internal validity of our findings.

Supplementary Table S1. Clinical characteristics in the Radiopathomics analysis set, TCGA validation set, CPTAC validation set, and Radiopathomics test set.

Characteristics	Radiopathomics analysis set			TCGA validation set			CPTAC validation set			Radiopathomics test set		
	High-grade (N=115)	Non-high-grade (N=149)	P-value	High-grade (N=257)	Non-high-grade (N=150)	P-value	High-grade (N=153)	Non-high-grade (N=62)	P-value	High-grade (N=22)	Non-high-grade (N=80)	P-value
**Age(years)**												
Mean ± SD	62.0±10.5	60.1±10.1	0.120	64.7±10.0	65.7±9.33	0.372	61.5±8.65	68.3±8.29	<0.001	61.5±12.3	60.6±10.7	0.794
**Gender**												
Male	50 (43.5%)	43 (28.9%)	0.019	128 (49.8%)	58 (38.7%)	0.031	99 (64.7%)	36 (58.1%)	0.436	13 (59.1%)	24 (30.0%)	0.023
Female	65 (56.5%)	106 (71.1%)		129 (50.2%)	92 (61.3%)		54 (35.3%)	26 (41.9%)		9 (40.9%)	56 (70.0%)	

P value < 0.05 was considered statistically significance.

Supplementary Table S2. Clinical characteristics in the Radiopathomics training set and Hold-out set.

Characteristics	Radiopathomics training set			Hold-out set		
	High-grade (N=80)	Non-high-grade (N=105)	P-value	High-grade (N=35)	Non-high-grade (N=44)	P-value
**Age(years)**						
Mean ± SD	62.0±10.0	59.4±10.1	0.072	62.1±11.8	62.0±9.95	0.886
**Gender**						
Male	36 (45.0%)	34 (32.4%)	0.110	14 (40.0%)	9 (20.5%)	0.099
Female	44 (55.0%)	71 (67.6%)		21 (60.0%)	35 (79.5%)	

P value < 0.05 was considered statistically signifificance.

Supplementary Table S3. Clinical characteristics in RPG training set, RPG-TCGA validation set, and RPG-CPTAC validation set.

Characteristics	RPG training set			RPG-TCGA validation set			RPG-CPTAC validation set		
	High-grade (N=10)	Non-high-grade (N=26)	P-value	High-grade (N=14)	Non-high-grade (N=10)	P-value	High-grade (N=12)	Non-high-grade (N=6)	P-value
**Age(years)**									
Mean ± SD	64.5±12.9	57.8±10.7	0.128	62.9±9.94	72.0±5.68	0.022	67.3±9.85	66.8±5.38	0.638
**Gender**									
Male	2 (20.0%)	8 (30.8%)	0.689	9 (64.3%)	5 (50.0%)	0.678	3 (25.0%)	0 (0%)	0.515
Female	8 (80.0%)	18 (69.2%)		5 (35.7%)	5 (50.0%)		9 (75.0%)	6 (100%)	

P value < 0.05 was considered statistically signifificance.

Supplementary Table S4. Coefficient weights for each category of features retained by LASSO.

Feature category	Variables	Feature name	Coefficient weight
Handcrafted pathomics features	HPF_2	StDev_IdentifySecondaryObjects_AreaShape_Zernike_5_5	0.209695
	HPF_3	StDev_IdentifySecondaryObjects_Location_MaxIntensity_Y_Hematoxylin	0.050132
	HPF_4	StDev_IdentifySecondaryObjects_Texture_SumEntropy_Hematoxylin_3_02_256	-0.01144
	HPF_5	Median_IdentifySecondaryObjects_AreaShape_Zernike_1_1	-0.03086
	HPF_1	StDev_IdentifySecondaryObjects_AreaShape_Zernike_2_2	-0.03145
Handcrafted radiomics features	HRF_4	wavelet-LLL_glszm_SmallAreaHighGrayLevelEmphasis	0.273869
	HRF_5	original_firstorder_Minimum	0.092721
	HRF_3	log-sigma-3-0-mm-3D_glcm_Autocorrelation	-0.01382
	HRF_1	log-sigma-2-0-mm-3D_glcm_InverseVariance	-0.0415
	HRF_2	log-sigma-3-0-mm-3D_firstorder_10Percentile	-0.06153
DL pathomics features	DLPF_8	prob0906	0.083053
	DLPF_1	prob0518	0.064152
	DLPF_11	prob0957	0.060557
	DLPF_2	prob0533	0.057739
	DLPF_13	prob0966	0.055005
	DLPF_4	prob071	0.048679
	DLPF_7	prob0886	0.047206
	DLPF_14	prob0986	0.033777
	DLPF_5	prob0776	0.028814
	DLPF_10	prob0945	0.02832
	DLPF_3	prob0627	0.020461
	DLPF_6	prob0802	0.019191
	DLPF_12	prob0958	0.010762
	DLPF_9	prob0917	0.00614
DL radiomics features	DLRF_9	RESNET_424	0.105602
	DLRF_2	RESNET_81	0.060364
	DLRF_11	RESNET_443	0.048517
	DLRF_1	RESNET_20	0.031902
	DLRF_10	RESNET_425	-0.00272
	DLRF_5	RESNET_283	-0.01291
	DLRF_6	RESNET_387	-0.01373
	DLRF_4	RESNET_202	-0.02689
	DLRF_8	RESNET_411	-0.04728
	DLRF_3	RESNET_199	-0.05079
	DLRF_7	RESNET_391	-0.07544

DL, deep learning; DLPF, deep learning pathomics features; DLRF, deep learning radiomics features; HRF, handcrafted radiomics features; HPF, handcrafted pathomics features.

Supplementary Table S5. Performance comparison between the DHRPs model and pathologists at matched operating points.

	Sensitivity		p-value	Specificity		p-value
	Pathologist	Model (95% CI)		Pathologist	Model (95% CI)	
Pathologist 1	0.727	0.864 (0.696–1.000)	0.180	0.825	0.938 (0.880–0.987)	0.007
Pathologist 2	0.682	0.727 (0.500–0.909)	0.739	0.912	0.938 (0.880–0.987)	0.480

Supplementary Table S6. The Zsummary module preservation results between RPG training set and TCGA validation set.

MergeModule	MedianRank.pres	MedianRank.qual	Zsummary.pres	Zsummary.qual	Number of genes
black	4	5	25	39	198
blue	9	9	22	45	943
brown	3	8	38	34	316
green	5	6.5	21	40	273
magenta	6	2	8.3	30	97
pink	9	3	5.6	28	104
purple	1	1	13	17	33
red	2	4	32	46	252
turquoise	10	10	19	41	1826
yellow	6	6.5	23	44	309

The Zsummary module preservation results between RPG training set and TCGA validation set. The gray and gold modules were excluded.

Supplementary Table S7. The Zsummary module preservation results between RPG training set and CPTAC validation set.

MergeModule	MedianRank.pres	MedianRank.qual	Zsummary.pres	Zsummary.qual	Number of genes
black	5	5	28	39	198
blue	10	9	17	45	943
brown	3	8	48	34	316
green	5	6.5	27	40	273
magenta	6	2	9.3	30	97
pink	8	3	6.5	28	104
purple	1	1	17	17	33
red	3	4	39	46	252
turquoise	10	10	22	41	1826
yellow	6	6.5	22	44	309

The Zsummary module preservation results between RPG training set and CPTAC validation set. The gray and gold modules were excluded.

Supplementary Table S8. A summary of the genes in the two reproducible modules significantly associated with IPA grading.

Module name	Gene name
Green	YBX2,GGCT,SCIN,E2F2,ANLN,BRCA1,GCLM,DEPDC1,IBSP,DEPDC1B,HOXC8,CP,TNFRSF9,RAD51,POLQ,DCBLD2,SPAG4,MCM10,COL17A1,NGEF,ASPM,PFKP,PRR11,SDK2,SPTB,TRIP13,HLTF,MYO3B,SPP2,HMMR,MCM2,PPP2R3A,IPCEF1,GTSE1,UBE2T,PTPRH,NDC80,LRP2,OXCT1,ORC1,RAD54L,TMPRSS11E,TPX2,BIRC5,KIF4A,DLL3,ORC6,RGS17,CLSPN,CDC45,CDC6,BAMBI,GNPNAT1,CDKN3,GINS1,MYBL2,RAB5IF,EEF1A2,DNTTIP1,SMARCA1,PGK1,CENPI,NDRG1,PYCR3,MCM4,CKM,ASF1B,LIMK1,CA9,NEIL3,NCAPG,MDK,SLC35F2,FOXM1,RAD51AP1,VDR,TIMELESS,TTK,KIF20A,THBS4,LMNB1,PFKFB4,ECT2,CENPA,IGFBP2,KISS1R,CFHR3,SLC2A1,CDC20,STMN1,NEK2,CENPF,KIF14,TGFBI,NCAPH,KIF18A,DEPDC7,ZWINT,HOXC13,HOXC11,STIL,HJURP,WFDC3,PACSIN1,DLGAP5,EDN2,PLAAT1,TSPAN8,AUNIP,ADM2,PIMREG,TXNDC17,GINS2,KREMEN2,TOP2A,RHBG,DMGDH,BHMT2,EPHB2,CCNB1,MYCN,CDCA8,TCN1,MSI1,TROAP,ESPL1,SLC41A2,SCRN1,IGF2BP3,TTYH3,BRIP1,IL36RN,MMP13,KNL1,KIF11,CEP55,CENPE,GALNT6,GCNT3,TICRR,SLC16A3,HUNK,PLK4,KIF2C,NUF2,CRABP2,ANP32E,DTL,ATP8B2,PPFIA4,ABCA12,ACKR2,IL17RD,TPBG,CDCA5,NCAPG2,LRP12,MKI67,INCENP,SPC25,ADGRF1,GPR15,SKA1,GRIP1,BUB1B,CCNB2,PWWP3B,GAREM2,CDC25C,LY6K,RACGAP1,SPC24,SGO2,RPL39L,CDC25A,MAD2L1,ITGA2,RHOBTB3,IL22RA2,PTTG1,SLC29A4,PDP1,MELK,PGM2L1,SKA3,E2F7,PCLAF,CDT1,CHAF1A,TK1,PBK,TSPAN5,ADRB2,GPRIN1,CKAP2L,BUB1,CHRNA5,ADRA1B,CDK1,METTL7B,NPAS2,EMB,GPR37,CDCA4,SHCBP1,ESCO2,ANO5,RRM2,FRMD5,C11orf86,EXO1,IL20RB,ASPHD1,UBE2C,MARCKSL1,ACBD7,LMNB2,TYMS,ZNF518B,DNAJC22,C5orf46,RFLNA,AURKB,HOXC9,HOXC10,NXPH4,KPNA2,GLUD2,ABAT,CABCOCO1,GBP6,IQGAP3,CDCA2,DRD1,KCNQ5,KIF18B,SAPCD2,GLDN,HPDL,NCCRP1,FAM111B,WNK3,SCN8A,HCAR1,FAM3C,BLM,OGDHL,SLC28A3,HOXC6,ERO1A,HOXC4,KRTAP4-1,B3GNT6,WDHD1,APCDD1L,H2BC18,ZYG11A,PTPN20,PSORS1C1,SERPINB5,MAP10,GPC2,TMEM150C,PRKDC,PCDHGA10,MEX3A,RNF225,H2BC8,UHRF1,H2AC16,H2BC7,RDM1,H3C10
Yellow	CFH,MCUB,MEOX1,ITGA3,CACNA2D2,SELE,CYTH3,FMO1,DPEP1,VSIG2,HSD17B6,VCAN,EPHA3,XK,CHRDL2,LAMC2,COL11A1,PRKCZ,SCT,PFN2,NTN4,SEMA3C,FAP,MOXD1,KIF3C,FOLH1,NOX4,MMP2,NID2,PTHLH,CPXM1,FXYD3,CCDC80,IL11,SMIM24,PGC,PCSK5,NRP1,PRTFDC1,IGFALS,MMP11,SUSD2,PLA2G3,SLC5A1,HIF1A,DOK5,BMP7,ISM1,SYNDIG1,MXRA5,TNFSF13B,MEDAG,XYLT1,PDGFRL,CCN4,STMN2,SLC39A14,OLFM2,MYH14,MEIS3,CLEC11A,PLEKHA4,COMP,DLX5,COBL,PCOLCE,SFRP4,TSPAN13,GLI3,AEBP1,AK1,RASD1,COL1A1,MAP2K6,CRACD,FOLR1,P3H3,ELK3,ADGRD1,MAN1A1,SMOC2,LOX,SPARC,RASGRF2,DPYSL3,STC2,IFT57,AADAC,FNDC4,PDE1A,FN1,IGFBP5,FHL2,PRRX1,GPX7,MFAP2,RGS4,PLPPR4,PPL,PGF,TGFB3,PLXDC2,CRISPLD1,CPXM2,INHBA,BICC1,BHLHE41,IL13RA2,COL10A1,TNFAIP6,SNAI1,PMEPA1,PTGER2,IL37,C3,LAMP5,OMD,ISLR,LOXL1,AJUBA,PLVAP,OLFM1,COL5A1,GALNT15,GFPT2,PODNL1,MATN3,SERPINF1,MATN2,DCLK1,POSTN,RNF128,SFTPD,LOXL2,PEBP4,NAV1,IL2RA,DAGLA,LCA5,HTR2B,ITM2C,SERPINE2,ALDH1L2,PLXNC1,NKX2-8,ABHD17C,ANGPTL2,ALDH1B1,TUBB2A,TUBB2B,PI15,SULF1,ITGA11,DUOX1,CGREF1,CYP1B1,ITGAV,CILP,ENPEP,LEF1,ERP27,LUM,FRMD6,ARMH4,ARRDC4,CDH11,FKBP10,MISP3,CYP4B1,CTSK,FAM171B,ITGA9,COL8A1,BOC,CORIN,SFRP2,ADAMTS16,IGFBP3,PAMR1,P4HA3,GGTLC1,ADAMTS12,BEND6,KCNE4,SPOCK1,LGI2,THY1,UBASH3B,SLC16A1,TIAM1,FBXO32,STEAP2,STC1,C1R,LRRC36,CILP2,PDZK1IP1,RBP7,DHRS3,MXRA8,VCAM1,KIF26B,FRZB,SLC16A14,COL6A3,SLC15A2,FBLN2,RPL22L1,TM4SF18,GASK1B,F2RL2,GPX8,EGFLAM,TMEM200A,STEAP1,COL1A2,FNDC1,ADCY1,CTHRC1,GJB2,DDIAS,DACT1,HTRA1,GPR176,FBN1,CLMP,CYB5A,NNMT,SCNN1G,SCG5,GREM1,CERCAM,RAB3IL1,DDIT4,COL3A1,ROR2,MUC15,PCDH7,ROBO1,CST2,CST1,IRX1,HTRA3,PDGFD,CDC42BPG,KRT19,TPPP,PDE7B,AQP4,SCG2,LRRC15,MAB21L4,PPP1R3B,NET1,PODN,SH3PXD2B,P2RY2,SUGCT,B3GNT5,BASP1,NIM1K,CD163L1,CD28,MSC,LDLRAD3,FJX1,BHLHE22,F2R,TMEM45A,GPR3,HHIPL1,BACE2,C1S,TSHZ2,BGN,TMEM119,ALDH1A3,FAM174B,THBS2,C5orf38,BCAM,COL14A1,PLA2G2A,MAOA,NUGGC,S100A14,SPATS2L,SULF2,LAMA2,ALKAL1,VEPH1,FAM177B,ITGBL1,SMOC1,ZNF521,GPRIN2,COL5A2,COL15A1,ERICH2,DIO2,MLLT11,ANG,TAFA5,SLC12A8,BTBD19,TMEM238,SMIM3,HOXB7,MILR1

Supplementary Table S9. Power to detect significant associations for green and yellow module eigengenes.

Module	Subset	Sample_Size (n)	Correlation (r)	Statistical_Power
Green	RPG training set	36	0.42	0.740
Green	TCGA validation set	407	0.39	1.000
Green	CPTAC validation set	215	0.54	1.000
Yellow	RPG training set	36	0.34	0.539
Yellow	TCGA validation set	407	0.14	0.809
Yellow	CPTAC validation set	215	0.35	0.999

Supplementary Table S10. Top five biological pathways most enriched in the green and yellow gene modules of the RPG training set.

Module	Pathway	GeneRatio	pvalue	FDR	qvalue	Genes	Count	Correlation	Database
Green	GOBP_CHROMOSOME_SEGREGATION	48/261	2.32E-34	6.36E-31	5.83E-31	BRCA1/TRIP13/NDC80/KIF4A/CDC6/NCAPG/TTK/ECT2/CDC20/NEK2/CENPF/KIF14/NCAPH/KIF18A/ZWINT/HJURP/DLGAP5/TOP2A/CCNB1/CDCA8/ESPL1/BRIP1/KNL1/CEP55/CENPE/KIF2C/NUF2/CDCA5/NCAPG2/MKI67/INCENP/SPC25/SKA1/BUB1B/RACGAP1/SPC24/SGO2/MAD2L1/PTTG1/SKA3/CDT1/BUB1/ESCO2/UBE2C/AURKB/CDCA2/KIF18B/MAP10	48	Positive	GO
	GOBP_NUCLEAR_CHROMOSOME_SEGREGATION	42/261	3.97E-31	5.43E-28	4.98E-28	TRIP13/NDC80/KIF4A/CDC6/NCAPG/TTK/ECT2/CDC20/NEK2/CENPF/KIF14/NCAPH/KIF18A/ZWINT/DLGAP5/TOP2A/CCNB1/CDCA8/ESPL1/BRIP1/KNL1/CEP55/CENPE/KIF2C/NUF2/CDCA5/NCAPG2/INCENP/SPC25/BUB1B/RACGAP1/SPC24/SGO2/MAD2L1/PTTG1/CDT1/BUB1/ESCO2/UBE2C/AURKB/KIF18B/MAP10	42	Positive	GO
	GOBP_MITOTIC_SISTER_CHROMATID_SEGREGATION	34/261	7.32E-30	6.69E-27	6.13E-27	TRIP13/NDC80/KIF4A/CDC6/NCAPG/TTK/CDC20/NEK2/CENPF/KIF14/NCAPH/KIF18A/ZWINT/DLGAP5/CCNB1/CDCA8/ESPL1/CEP55/CENPE/KIF2C/NUF2/CDCA5/NCAPG2/BUB1B/RACGAP1/SGO2/MAD2L1/PTTG1/CDT1/BUB1/UBE2C/AURKB/KIF18B/MAP10	34	Positive	GO
	HALLMARK_G2M_CHECKPOINT	47/113	5.29E-35	2.06E-33	1.84E-33	E2F2/POLQ/HMMR/MCM2/NDC80/RAD54L/TPX2/BIRC5/KIF4A/ORC6/CDC45/CDC6/CDKN3/MYBL2/TTK/LMNB1/CENPA/CDC20/STMN1/NEK2/CENPF/STIL/GINS2/TOP2A/TROAP/ESPL1/KNL1/KIF11/CENPE/PLK4/KIF2C/MKI67/INCENP/CCNB2/RACGAP1/CDC25A/MAD2L1/PTTG1/CHAF1A/PBK/BUB1/CDK1/EXO1/UBE2C/AURKB/HOXC10/KPNA2	47	Positive	HALLMARK
	HALLMARK_E2F_TARGETS	44/113	3.09E-31	6.03E-30	5.37E-30	BRCA1/DEPDC1/TRIP13/HMMR/MCM2/UBE2T/BIRC5/KIF4A/ORC6/CDKN3/GINS1/MYBL2/MCM4/ASF1B/RAD51AP1/TIMELESS/LMNB1/CDC20/STMN1/DLGAP5/TOP2A/CDCA8/ESPL1/CENPE/PLK4/KIF2C/ANP32E/MKI67/SPC25/BUB1B/CCNB2/RACGAP1/SPC24/CDC25A/MAD2L1/PTTG1/MELK/TK1/CDK1/RRM2/AURKB/KPNA2/KIF18B/PRKDC	44	Positive	HALLMARK
Yellow	GOCC_COLLAGEN_CONTAINING_EXTRACELLULAR_MATRIX	50/239	2.24E-34	6.81E-31	5.41E-31	VCAN/COL11A1/MMP2/NID2/CCDC80/BMP7/MXRA5/COMP/PCOLCE/AEBP1/COL1A1/SMOC2/SPARC/FN1/MFAP2/TGFB3/COL10A1/OMD/LOXL1/COL5A1/MATN3/SERPINF1/MATN2/POSTN/LOXL2/SERPINE2/ANGPTL2/SULF1/CILP/LUM/COL8A1/SFRP2/COL6A3/FBLN2/EGFLAM/COL1A2/CTHRC1/HTRA1/FBN1/GREM1/COL3A1/LRRC15/PODN/BGN/THBS2/COL14A1/LAMA2/SMOC1/COL5A2/COL15A1	50	Positive	GO
	GOMF_EXTRACELLULAR_MATRIX_STRUCTURAL_CONSTITUENT	32/239	1.37E-28	2.09E-25	1.66E-25	VCAN/COL11A1/NID2/MXRA5/COMP/PCOLCE/AEBP1/COL1A1/SPARC/FN1/MFAP2/COL10A1/COL5A1/MATN3/MATN2/POSTN/CILP/LUM/COL8A1/COL6A3/FBLN2/COL1A2/CTHRC1/FBN1/COL3A1/PODN/BGN/THBS2/COL14A1/LAMA2/COL5A2/COL15A1	32	Positive	GO

	GOBP_EXTERNAL_ENCAPSULATING_STRUCTURE_ORGANIZATION	36/239	1.64E-24	1.67E-21	1.33E-21	COL11A1/FAP/MMP2/NID2/CCDC80/MMP11/COMP/AEBP1/COL1A1/SMOC2/LOX/COL10A1/LOXL1/COL5A1/POSTN/LOXL2/SULF1/CYP1B1/LUM/FKBP10/CTSK/COL8A1/SFRP2/ADAMTS16/ADAMTS12/FBLN2/EGFLAM/COL1A2/GREM1/COL3A1/SH3PXD2B/COL14A1/SULF2/SMOC1/COL5A2/COL15A1	36	Positive	GO
	REACTOME_EXTRACELLULAR_MATRIX_ORGANIZATION	40/146	7.67E-29	2.98E-26	2.60E-26	VCAN/COL11A1/MMP2/NID2/MMP11/BMP7/COMP/PCOLCE/COL1A1/P3H3/LOX/SPARC/FN1/MFAP2/TGFB3/COL10A1/LOXL1/COL5A1/MATN3/LOXL2/ITGA11/ITGAV/LUM/CTSK/ITGA9/COL8A1/ADAMTS16/P4HA3/VCAM1/COL6A3/FBLN2/COL1A2/HTRA1/FBN1/COL3A1/BGN/COL14A1/LAMA2/COL5A2/COL15A1	40	Positive	REACTOME
	HALLMARK_EPITHELIAL_MESENCHYMAL_TRANSITION	51/110	4.97E-41	2.04E-39	1.83E-39	VCAN/COL11A1/PFN2/FAP/MMP2/NID2/PTHLH/MXRA5/COMP/PCOLCE/SFRP4/COL1A1/LOX/SPARC/DPYSL3/FN1/PRRX1/GPX7/RGS4/INHBA/PMEPA1/LOXL1/COL5A1/MATN3/MATN2/POSTN/LOXL2/SERPINE2/ITGAV/LUM/CDH11/IGFBP3/SPOCK1/THY1/VCAM1/COL6A3/FBLN2/COL1A2/CTHRC1/HTRA1/FBN1/NNMT/GREM1/COL3A1/SCG2/LRRC15/BASP1/BGN/THBS2/LAMA2/COL5A2	51	Positive	HALLMARK

Each module was categorized into two subsets of genes that were positively or negatively correlated with the module eigengene (ME).

Supplementary Table S11. Top five biological pathways most enriched in the green and yellow gene modules of the TCGA validation set.

Module	Pathway	GeneRatio	pvalue	FDR	qvalue	Genes	Count	Correlation	Database
Green	GOBP_CHROMOSOME_SEGREGATION	48/239	3.03E-36	7.67E-33	6.99E-33	BRCA1/TRIP13/NDC80/KIF4A/CDC6/NCAPG/TTK/ECT2/CDC20/NEK2/CENPF/KIF14/NCAPH/KIF18A/ZWINT/HJURP/DLGAP5/TOP2A/CCNB1/CDCA8/ESPL1/BRIP1/KNL1/CEP55/CENPE/KIF2C/NUF2/CDCA5/NCAPG2/MKI67/INCENP/SPC25/SKA1/BUB1B/RACGAP1/SPC24/SGO2/MAD2L1/PTTG1/SKA3/CDT1/BUB1/ESCO2/UBE2C/AURKB/CDCA2/KIF18B/MAP10	48	Positive	GO
	GOBP_NUCLEAR_CHROMOSOME_SEGREGATION	42/239	9.23E-33	1.17E-29	1.07E-29	TRIP13/NDC80/KIF4A/CDC6/NCAPG/TTK/ECT2/CDC20/NEK2/CENPF/KIF14/NCAPH/KIF18A/ZWINT/DLGAP5/TOP2A/CCNB1/CDCA8/ESPL1/BRIP1/KNL1/CEP55/CENPE/KIF2C/NUF2/CDCA5/NCAPG2/INCENP/SPC25/BUB1B/RACGAP1/SPC24/SGO2/MAD2L1/PTTG1/CDT1/BUB1/ESCO2/UBE2C/AURKB/KIF18B/MAP10	42	Positive	GO
	GOBP_MITOTIC_SISTER_CHROMATID_SEGREGATION	34/239	3.45E-31	2.53E-28	2.31E-28	TRIP13/NDC80/KIF4A/CDC6/NCAPG/TTK/CDC20/NEK2/CENPF/KIF14/NCAPH/KIF18A/ZWINT/DLGAP5/CCNB1/CDCA8/ESPL1/CEP55/CENPE/KIF2C/NUF2/CDCA5/NCAPG2/BUB1B/RACGAP1/SGO2/MAD2L1/PTTG1/CDT1/BUB1/UBE2C/AURKB/KIF18B/MAP10	34	Positive	GO
	GOBP_MITOTIC_NUCLEAR_DIVISION	41/239	3.99E-31	2.53E-28	2.31E-28	ANLN/TRIP13/NDC80/TPX2/KIF4A/CDC6/MYBL2/NCAPG/TTK/CDC20/NEK2/CENPF/KIF14/NCAPH/KIF18A/ZWINT/DLGAP5/CCNB1/CDCA8/ESPL1/KIF11/CEP55/CENPE/KIF2C/NUF2/CDCA5/NCAPG2/MKI67/BUB1B/CDC25C/RACGAP1/SGO2/MAD2L1/PTTG1/CDT1/BUB1/UBE2C/AURKB/CDCA2/KIF18B/MAP10	41	Positive	GO
	HALLMARK_G2M_CHECKPOINT	47/107	2.36E-36	8.96E-35	7.94E-35	E2F2/POLQ/HMMR/MCM2/NDC80/RAD54L/TPX2/BIRC5/KIF4A/ORC6/CDC45/CDC6/CDKN3/MYBL2/TTK/LMNB1/CENPA/CDC20/STMN1/NEK2/CENPF/STIL/GINS2/TOP2A/TROAP/ESPL1/KNL1/KIF11/CENPE/PLK4/KIF2C/MKI67/INCENP/CCNB2/RACGAP1/CDC25A/MAD2L1/PTTG1/CHAF1A/PBK/BUB1/CDK1/EXO1/UBE2C/AURKB/HOXC10/KPNA2	47	Positive	HALLMARK
Yellow	GOCC_COLLAGEN_CONTAINING_EXTRACELLULAR_MATRIX	52/250	1.34E-35	4.20E-32	3.36E-32	VCAN/LAMC2/COL11A1/NTN4/MMP2/NID2/CCDC80/BMP7/MXRA5/COMP/PCOLCE/AEBP1/COL1A1/SMOC2/SPARC/FN1/MFAP2/TGFB3/COL10A1/OMD/LOXL1/COL5A1/MATN3/SERPINF1/MATN2/POSTN/LOXL2/SERPINE2/ANGPTL2/SULF1/CILP/LUM/COL8A1/SFRP2/COL6A3/FBLN2/EGFLAM/COL1A2/CTHRC1/HTRA1/FBN1/GREM1/COL3A1/LRRC15/PODN/BGN/THBS2/COL14A1/LAMA2/SMOC1/COL5A2/COL15A1	52	Positive	GO
	GOMF_EXTRACELLULAR_MATRIX_STRUCTURAL_CONSTITUENT	33/250	2.87E-29	4.50E-26	3.59E-26	VCAN/LAMC2/COL11A1/NID2/MXRA5/COMP/PCOLCE/AEBP1/COL1A1/SPARC/FN1/MFAP2/COL10A1/COL5A1/MATN3/MATN2/POSTN/CILP/LUM/COL8A1/COL6A3/FBLN2/COL1A2/CTHRC1/FBN1/COL3A1/PODN/BGN/THBS2/COL14A1/LAMA2/COL5A2/COL15A1	33	Positive	GO

	GOBP_EXTERNAL_ENCAPSULATING_STRUCTURE_ORGANIZATION	37/250	6.86E-25	7.16E-22	5.72E-22	COL11A1/NTN4/FAP/MMP2/NID2/CCDC80/MMP11/COMP/AEBP1/COL1A1/SMOC2/LOX/COL10A1/LOXL1/COL5A1/POSTN/LOXL2/SULF1/CYP1B1/LUM/FKBP10/CTSK/COL8A1/SFRP2/ADAMTS16/ADAMTS12/FBLN2/EGFLAM/COL1A2/GREM1/COL3A1/SH3PXD2B/COL14A1/SULF2/SMOC1/COL5A2/COL15A1	37	Positive	GO
	REACTOME_EXTRACELLULAR_MATRIX_ORGANIZATION	43/156	4.31E-31	1.72E-28	1.51E-28	ITGA3/VCAN/LAMC2/COL11A1/NTN4/MMP2/NID2/MMP11/BMP7/COMP/PCOLCE/COL1A1/P3H3/LOX/SPARC/FN1/MFAP2/TGFB3/COL10A1/LOXL1/COL5A1/MATN3/LOXL2/ITGA11/ITGAV/LUM/CTSK/ITGA9/COL8A1/ADAMTS16/P4HA3/VCAM1/COL6A3/FBLN2/COL1A2/HTRA1/FBN1/COL3A1/BGN/COL14A1/LAMA2/COL5A2/COL15A1	43	Positive	REACTOME
	HALLMARK_EPITHELIAL_MESENCHYMAL_TRANSITION	52/116	6.53E-41	2.68E-39	2.34E-39	VCAN/LAMC2/COL11A1/PFN2/FAP/MMP2/NID2/PTHLH/MXRA5/COMP/PCOLCE/SFRP4/COL1A1/LOX/SPARC/DPYSL3/FN1/PRRX1/GPX7/RGS4/INHBA/PMEPA1/LOXL1/COL5A1/MATN3/MATN2/POSTN/LOXL2/SERPINE2/ITGAV/LUM/CDH11/IGFBP3/SPOCK1/THY1/VCAM1/COL6A3/FBLN2/COL1A2/CTHRC1/HTRA1/FBN1/NNMT/GREM1/COL3A1/SCG2/LRRC15/BASP1/BGN/THBS2/LAMA2/COL5A2	52	Positive	HALLMARK

Supplementary Table S12. Top five biological pathways most enriched in the green and yellow gene modules of the CPTAC validation set.

Module	Pathway	GeneRatio	pvalue	FDR	qvalue	Genes	Count	Correlation	Database
Green	GOBP_CHROMOSOME_SEGREGATION	48/234	1.05E-36	2.67E-33	2.43E-33	BRCA1/TRIP13/NDC80/KIF4A/CDC6/NCAPG/TTK/ECT2/CDC20/NEK2/CENPF/KIF14/NCAPH/KIF18A/ZWINT/HJURP/DLGAP5/TOP2A/CCNB1/CDCA8/ESPL1/BRIP1/KNL1/CEP55/CENPE/KIF2C/NUF2/CDCA5/NCAPG2/MKI67/INCENP/SPC25/SKA1/BUB1B/RACGAP1/SPC24/SGO2/MAD2L1/PTTG1/SKA3/CDT1/BUB1/ESCO2/UBE2C/AURKB/CDCA2/KIF18B/MAP10	48	Positive	GO
	GOBP_NUCLEAR_CHROMOSOME_SEGREGATION	42/234	3.71E-33	4.69E-30	4.27E-30	TRIP13/NDC80/KIF4A/CDC6/NCAPG/TTK/ECT2/CDC20/NEK2/CENPF/KIF14/NCAPH/KIF18A/ZWINT/DLGAP5/TOP2A/CCNB1/CDCA8/ESPL1/BRIP1/KNL1/CEP55/CENPE/KIF2C/NUF2/CDCA5/NCAPG2/INCENP/SPC25/BUB1B/RACGAP1/SPC24/SGO2/MAD2L1/PTTG1/CDT1/BUB1/ESCO2/UBE2C/AURKB/KIF18B/MAP10	42	Positive	GO
	GOBP_MITOTIC_SISTER_CHROMATID_SEGREGATION	34/234	1.65E-31	1.04E-28	9.50E-29	TRIP13/NDC80/KIF4A/CDC6/NCAPG/TTK/CDC20/NEK2/CENPF/KIF14/NCAPH/KIF18A/ZWINT/DLGAP5/CCNB1/CDCA8/ESPL1/CEP55/CENPE/KIF2C/NUF2/CDCA5/NCAPG2/BUB1B/RACGAP1/SGO2/MAD2L1/PTTG1/CDT1/BUB1/UBE2C/AURKB/KIF18B/MAP10	34	Positive	GO
	GOBP_MITOTIC_NUCLEAR_DIVISION	41/234	1.65E-31	1.04E-28	9.50E-29	ANLN/TRIP13/NDC80/TPX2/KIF4A/CDC6/MYBL2/NCAPG/TTK/CDC20/NEK2/CENPF/KIF14/NCAPH/KIF18A/ZWINT/DLGAP5/CCNB1/CDCA8/ESPL1/KIF11/CEP55/CENPE/KIF2C/NUF2/CDCA5/NCAPG2/MKI67/BUB1B/CDC25C/RACGAP1/SGO2/MAD2L1/PTTG1/CDT1/BUB1/UBE2C/AURKB/CDCA2/KIF18B/MAP10	41	Positive	GO
	HALLMARK_G2M_CHECKPOINT	47/108	4.03E-36	1.57E-34	1.40E-34	E2F2/POLQ/HMMR/MCM2/NDC80/RAD54L/TPX2/BIRC5/KIF4A/ORC6/CDC45/CDC6/CDKN3/MYBL2/TTK/LMNB1/CENPA/CDC20/STMN1/NEK2/CENPF/STIL/GINS2/TOP2A/TROAP/ESPL1/KNL1/KIF11/CENPE/PLK4/KIF2C/MKI67/INCENP/CCNB2/RACGAP1/CDC25A/MAD2L1/PTTG1/CHAF1A/PBK/BUB1/CDK1/EXO1/UBE2C/AURKB/HOXC10/KPNA2	47	Positive	HALLMARK
Yellow	GOCC_COLLAGEN_CONTAINING_EXTRACELLULAR_MATRIX	50/235	9.39E-35	2.87E-31	2.28E-31	VCAN/LAMC2/COL11A1/MMP2/NID2/CCDC80/BMP7/MXRA5/COMP/PCOLCE/AEBP1/COL1A1/SMOC2/SPARC/FN1/MFAP2/TGFB3/COL10A1/OMD/LOXL1/COL5A1/MATN3/SERPINF1/POSTN/LOXL2/SERPINE2/ANGPTL2/SULF1/CILP/LUM/COL8A1/SFRP2/COL6A3/FBLN2/EGFLAM/COL1A2/CTHRC1/HTRA1/FBN1/GREM1/COL3A1/LRRC15/PODN/BGN/THBS2/COL14A1/LAMA2/SMOC1/COL5A2/COL15A1	50	Positive	GO
	GOMF_EXTRACELLULAR_MATRIX_STRUCTURAL_CONSTITUENT	32/235	7.90E-29	1.21E-25	9.58E-26	VCAN/LAMC2/COL11A1/NID2/MXRA5/COMP/PCOLCE/AEBP1/COL1A1/SPARC/FN1/MFAP2/COL10A1/COL5A1/MATN3/POSTN/CILP/LUM/COL8A1/COL6A3/FBLN2/COL1A2/CTHRC1/FBN1/COL3A1/PODN/BGN/THBS2/COL14A1/LAMA2/COL5A2/COL15A1	32	Positive	GO

	GOBP_EXTERNAL_ENCAPSULATING_STRUCTURE_ORGANIZATION	36/235	9.02E-25	9.18E-22	7.29E-22	COL11A1/FAP/MMP2/NID2/CCDC80/MMP11/COMP/AEBP1/COL1A1/SMOC2/LOX/COL10A1/LOXL1/COL5A1/POSTN/LOXL2/SULF1/CYP1B1/LUM/FKBP10/CTSK/COL8A1/SFRP2/ADAMTS16/ADAMTS12/FBLN2/EGFLAM/COL1A2/GREM1/COL3A1/SH3PXD2B/COL14A1/SULF2/SMOC1/COL5A2/COL15A1	36	Positive	GO
	REACTOME_EXTRACELLULAR_MATRIX_ORGANIZATION	41/147	6.82E-30	2.69E-27	2.35E-27	ITGA3/VCAN/LAMC2/COL11A1/MMP2/NID2/MMP11/BMP7/COMP/PCOLCE/COL1A1/P3H3/LOX/SPARC/FN1/MFAP2/TGFB3/COL10A1/LOXL1/COL5A1/MATN3/LOXL2/ITGA11/ITGAV/LUM/CTSK/COL8A1/ADAMTS16/P4HA3/VCAM1/COL6A3/FBLN2/COL1A2/HTRA1/FBN1/COL3A1/BGN/COL14A1/LAMA2/COL5A2/COL15A1	41	Positive	REACTOME
	HALLMARK_EPITHELIAL_MESENCHYMAL_TRANSITION	51/108	1.52E-41	6.23E-40	5.60E-40	VCAN/LAMC2/COL11A1/PFN2/FAP/MMP2/NID2/PTHLH/MXRA5/COMP/PCOLCE/SFRP4/COL1A1/LOX/SPARC/DPYSL3/FN1/PRRX1/GPX7/RGS4/INHBA/PMEPA1/LOXL1/COL5A1/MATN3/POSTN/LOXL2/SERPINE2/ITGAV/LUM/CDH11/IGFBP3/SPOCK1/THY1/VCAM1/COL6A3/FBLN2/COL1A2/CTHRC1/HTRA1/FBN1/NNMT/GREM1/COL3A1/SCG2/LRRC15/BASP1/BGN/THBS2/LAMA2/COL5A2	51	Positive	HALLMARK

Supplementary Table S13. Top 10 positively correlated and top 10 negatively correlated pathways from the GSEA enrichment analysis in the TCGA and CPTAC validation sets.

Correlation	Pathway	Core_enrichment	Set Size	NES(TCGA)	NES(CPTAC)
Positive	GOBP_CELL_CYCLE	ANLN/NCAPG/CDKN3/UBE2C/CENPA/FOXM1/DLGAP5/ESPL1/CDC20/E2F7/KIF4A/CENPE/TPX2/BUB1B/ASPM/EXO1/RRM2/PBK/MAD2L1/HJURP/NUF2/BIRC5/SLC16A1/KIF18B/CCNB1/NEK2/KIF14/MELK/ORC1/SKA1/KIF2C/RAD51AP1/AURKB/PLK4/MKI67/TOP2A/NCAPH/CDCA5/CCNB2/MYBL2/BUB1/CEP55/TTK/CDC45/KNL1/CDC25A/PRR11/PIMREG/TRIP13/CLSPN/CENPF/SKA3/CDCA2/CDK1/CDC6/RAD54L/NDC80/GTSE1/UHRF1/KIF20A/DDIAS/KIF11/CDC25C/RAD51/CDCA8/RACGAP1/AUNIP/TICRR/TUBB2B/GINS1/KIF18A/SAPCD2/SPC24/CDT1/MCM4/SPC25/ESCO2/SGO2/BRCA1/IQGAP3/PCLAF/ECT2/ZWINT/DTL/NCAPG2	112	3.472729	3.264672
	GOCC_CHROMOSOME	H2AC16/NCAPG/LOXL2/CENPA/FOXM1/E2F7/KIF4A/CENPE/BUB1B/MCM10/H2BC7/MAD2L1/HJURP/NUF2/BIRC5/CCNB1/NEK2/ORC1/SKA1/NEIL3/KIF2C/RAD51AP1/AURKB/PLK4/MKI67/TOP2A/NCAPH/POLQ/CDCA5/BUB1/TTK/CENPI/CDC45/KNL1/H2BC8/TRIP13/CENPF/SKA3/CDCA2/CDK1/NDC80/UHRF1/ORC6/GINS2/RAD51/CDCA8/SNAI1/AUNIP/GINS1/KIF18A/SPC24/CDT1/MCM4/SPC25/ESCO2/SGO2/BRCA1/ZWINT/DTL/NCAPG2/FAM111B/BLM/MCM2	100	2.778098	2.754381
	GOCC_SPINDLE	DLGAP5/ESPL1/CDC20/KIF4A/CENPE/TPX2/BUB1B/ASPM/MAD2L1/BIRC5/KIF18B/CCNB1/NEK2/KIF14/SKA1/KIF2C/AURKB/PLK4/CKAP2L/TTK/HMMR/CENPF/SKA3/CDK1/CDC6/SHCBP1/KIF20A/KIF11/CDCA8/RACGAP1/AUNIP	37	2.796613	2.789343
	GOBP_MITOTIC_CELL_CYCLE	ANLN/NCAPG/CDKN3/UBE2C/CENPA/FOXM1/DLGAP5/ESPL1/CDC20/E2F7/KIF4A/CENPE/TPX2/BUB1B/RRM2/PBK/MAD2L1/NUF2/KIF18B/CCNB1/NEK2/KIF14/MELK/ORC1/SKA1/KIF2C/AURKB/PLK4/MKI67/NCAPH/CDCA5/CCNB2/MYBL2/BUB1/CEP55/TTK/CDC45/CDC25A/TRIP13/CLSPN/CENPF/SKA3/CDCA2/CDK1/CDC6/NDC80/GTSE1/KIF20A/KIF11/CDC25C/RAD51/CDCA8/RACGAP1/TICRR/TUBB2B/GINS1/KIF18A/SAPCD2/CDT1/MCM4/SPC25/SGO2/BRCA1/IQGAP3/ECT2/ZWINT/DTL/NCAPG2/PTTG1/BLM/MCM2	83	3.430434	3.264303
	GOCC_CONDENSED_CHROMOSOME	NCAPG/CENPA/CENPE/BUB1B/MAD2L1/HJURP/NUF2/BIRC5/CCNB1/NEK2/SKA1/KIF2C/AURKB/MKI67/TOP2A/BUB1/TTK/CENPI/KNL1/CENPF/SKA3/NDC80/RAD51/KIF18A/SPC24/CDT1/SPC25/SGO2/BRCA1/ZWINT	32	2.766305	2.717789
	GOBP_CELL_CYCLE_PROCESS	ANLN/NCAPG/CDKN3/UBE2C/CENPA/FOXM1/DLGAP5/ESPL1/CDC20/E2F7/KIF4A/CENPE/TPX2/BUB1B/ASPM/RRM2/MAD2L1/NUF2/SLC16A1/KIF18B/CCNB1/NEK2/KIF14/MELK/ORC1/KIF2C/RAD51AP1/AURKB/PLK4/MKI67/TOP2A/NCAPH/CDCA5/CCNB2/MYBL2/BUB1/CEP55/TTK/CDC45/KNL1/CDC25A/TRIP13/CLSPN/CENPF/CDCA2/CDK1/CDC6/RAD54L/NDC80/GTSE1/KIF20A/KIF11/CDC25C/RAD51/CDCA8/RACGAP1/AUNIP/TICRR/GINS1/KIF18A/SAPCD2/SPC24/CDT1/MCM4/SPC25/ESCO2/SGO2/BRCA1/IQGAP3/PCLAF/ECT2/ZWINT/DTL/NCAPG2/PTTG1/BLM/MCM2	92	3.404094	3.138581
	GOBP_MITOTIC_CELL_CYCLE_PROCESS	ANLN/NCAPG/CDKN3/UBE2C/CENPA/FOXM1/DLGAP5/ESPL1/CDC20/E2F7/KIF4A/CENPE/TPX2/BUB1B/RRM2/MAD2L1/NUF2/KIF18B/CCNB1/NEK2/KIF14/MELK/ORC1/KIF2C/AURKB/MKI67/NCAPH/CDCA5/CCNB2/MYBL2/BUB1/CEP55/TTK/CDC45/CDC25A/TRIP13/CLSPN/CENPF/CDCA2/CDK1/CDC6/NDC80/GTSE1/KIF20A/KIF11/CDC25C/RAD51/CDCA8/RACGAP1/TICRR/GINS1/KIF18A/SAPCD2/CDT1/MCM4/SPC25/SGO2/BRCA1/IQGAP3/ECT2/ZWINT/DTL/NCAPG2/PTTG1/BLM/MCM2	75	3.423852	3.213663
	GOBP_ORGANELLE_FISSION	ANLN/NCAPG/UBE2C/DLGAP5/ESPL1/CDC20/KIF4A/CENPE/TPX2/BUB1B/ASPM/MAD2L1/NUF2/KIF18B/CCNB1/NEK2/KIF14/KIF2C/RAD51AP1/AURKB/MKI67/TOP2A/NCAPH/CDCA5/CCNB2/MYBL2/BUB1/CEP55/TTK/TRIP13/CENPF/CDCA2/CDC6/RAD54L/NDC80/KIF11/CDC25C/RAD51/CDCA8/RACGAP1/KIF18A/CDT1/SGO2/ZWINT/NCAPG2	52	3.007165	2.995214
	GOBP_CHROMOSOME_SEGREGATION	NCAPG/UBE2C/DLGAP5/ESPL1/CDC20/KIF4A/CENPE/BUB1B/MAD2L1/HJURP/NUF2/KIF18B/CCNB1/NEK2/KIF14/SKA1/KIF2C/AURKB/MKI67/TOP2A/NCAPH/CDCA5/BUB1/CEP55/TTK/KNL1/TRIP13/CENPF/SKA3/CDCA2/CDC6/NDC80/CDCA8/RACGAP1/KIF18A/SPC24/CDT1/SPC25/ESCO2/SGO2/BRCA1/ECT2/ZWINT/NCAPG2	48	3.061579	3.084141
	GOBP_CHROMOSOME_ORGANIZATION	NCAPG/LOXL2/UBE2C/CENPA/DLGAP5/ESPL1/CDC20/KIF4A/CENPE/BUB1B/H2BC7/EXO1/MAD2L1/HJURP/NUF2/KIF18B/CCNB1/NEK2/KIF14/KIF2C/AURKB/MKI67/TOP2A/NCAPH/POLQ/CDCA5/BUB1/CEP55/TTK/CENPI/CDC45/H2BC8/TRIP13/CENPF/CDK1/CDC6/RAD54L/NDC80/UHRF1/GINS2/RAD51/CDCA8/RACGAP1/SNAI1/GINS1/KIF18A/CDT1/MCM4/ESCO2/SGO2/BRCA1/ZWINT/NCAPG2/PTTG1/BLM/MCM2	67	3.201492	3.023976

	GOCC_CHROMOSOME_CENTROMERIC_REGION	NCAPG/CENPA/CENPE/BUB1B/MAD2L1/HJURP/NUF2/BIRC5/CCNB1/NEK2/SKA1/KIF2C/AURKB/TOP2A/CDCA5/BUB1/TTK/CENPI/KNL1/CENPF/SKA3/NDC80/CDCA8/SNAI1/KIF18A/SPC24/CDT1/SPC25/ESCO2/SGO2/ZWINT	32	2.840091	2.84358
	GOBP_MITOTIC_NUCLEAR_DIVISION	ANLN/NCAPG/UBE2C/DLGAP5/ESPL1/CDC20/KIF4A/CENPE/TPX2/BUB1B/MAD2L1/NUF2/KIF18B/CCNB1/NEK2/KIF14/KIF2C/AURKB/MKI67/NCAPH/CDCA5/MYBL2/BUB1/CEP55/TTK/TRIP13/CENPF/CDCA2/CDC6/NDC80/KIF11/CDC25C/CDCA8/RACGAP1/KIF18A/CDT1/SGO2/ZWINT/NCAPG2/PTTG1	43	2.910809	2.999279
	GOBP_MITOTIC_CELL_CYCLE_PHASE_TRANSITION	ANLN/CDKN3/UBE2C/FOXM1/DLGAP5/ESPL1/E2F7/CENPE/BUB1B/RRM2/MAD2L1/CCNB1/KIF14/MELK/ORC1/AURKB/CDCA5/CCNB2/BUB1/TTK/CDC25A/TRIP13/CLSPN/CENPF/CDK1/CDC6/NDC80/GTSE1/CDC25C/TICRR/CDT1/BRCA1/IQGAP3/ZWINT/DTL	40	2.947948	2.938841
	GOBP_NUCLEAR_CHROMOSOME_SEGREGATION	NCAPG/UBE2C/DLGAP5/ESPL1/CDC20/KIF4A/CENPE/BUB1B/MAD2L1/NUF2/KIF18B/CCNB1/NEK2/KIF14/KIF2C/AURKB/TOP2A/NCAPH/CDCA5/BUB1/CEP55/TTK/KNL1/TRIP13/CENPF/CDC6/NDC80/CDCA8/RACGAP1/KIF18A/SPC24/CDT1/SPC25/ESCO2/SGO2/ECT2/ZWINT/NCAPG2/PTTG1	42	2.921763	2.975726
	GOBP_REGULATION_OF_CELL_CYCLE	ANLN/CDKN3/UBE2C/FOXM1/DLGAP5/ESPL1/CDC20/E2F7/CENPE/TPX2/BUB1B/ASPM/RRM2/MAD2L1/BIRC5/CCNB1/NEK2/KIF14/ORC1/RAD51AP1/AURKB/PLK4/MKI67/CDCA5/CCNB2/BUB1/TTK/CDC45/CDC25A/PRR11/TRIP13/CLSPN/CENPF/CDCA2/CDK1/CDC6/NDC80/GTSE1/KIF20A/KIF11/CDC25C/RACGAP1/TICRR/CDT1/BRCA1/PCLAF/ECT2/ZWINT/DTL/PTTG1/BLM	67	2.841897	2.756656
	GOBP_SISTER_CHROMATID_SEGREGATION	NCAPG/UBE2C/DLGAP5/ESPL1/CDC20/KIF4A/CENPE/BUB1B/MAD2L1/NUF2/KIF18B/CCNB1/NEK2/KIF14/KIF2C/AURKB/TOP2A/NCAPH/CDCA5/BUB1/CEP55/TTK/TRIP13/CENPF/CDC6/NDC80/CDCA8/RACGAP1/KIF18A/CDT1/ESCO2/SGO2/ZWINT/NCAPG2/PTTG1	36	2.843825	2.969933
	GOBP_MITOTIC_SISTER_CHROMATID_SEGREGATION	NCAPG/UBE2C/DLGAP5/ESPL1/CDC20/KIF4A/CENPE/BUB1B/MAD2L1/NUF2/KIF18B/CCNB1/NEK2/KIF14/KIF2C/AURKB/NCAPH/CDCA5/BUB1/CEP55/TTK/TRIP13/CENPF/CDC6/NDC80/CDCA8/RACGAP1/KIF18A/CDT1/SGO2/ZWINT/NCAPG2/PTTG1	34	2.76571	2.896011
	GOBP_CELL_DIVISION	ANLN/NCAPG/UBE2C/CENPA/ESPL1/CDC20/E2F7/KIF4A/CENPE/TPX2/BUB1B/ASPM/MAD2L1/NUF2/KIF18B/CCNB1/NEK2/KIF14/SKA1/KIF2C/AURKB/PLK4/TOP2A/NCAPH/CDCA5/CCNB2/BUB1/CEP55/KNL1/CDC25A/PIMREG/CENPF/SKA3/CDCA2/CDK1/CDC6/NDC80/KIF20A/ORC6/KIF11/CDC25C/RACGAP1/SAPCD2/SPC24/CDT1/SPC25/SGO2/ECT2/ZWINT/NCAPG2/PTTG1/BLM	67	2.833969	2.786671
	GOBP_REGULATION_OF_MITOTIC_CELL_CYCLE_PHASE_TRANSITION	ANLN/UBE2C/DLGAP5/ESPL1/E2F7/CENPE/BUB1B/RRM2/MAD2L1/CCNB1/KIF14/ORC1/AURKB/CDCA5/BUB1/TTK/CDC25A/TRIP13/CLSPN/CENPF/CDK1/CDC6/NDC80/GTSE1/CDC25C/TICRR/CDT1/BRCA1/ZWINT/DTL/BLM	35	2.778863	2.788541
	GOCC_CHROMOSOMAL_REGION	NCAPG/CENPA/CENPE/BUB1B/MAD2L1/HJURP/NUF2/BIRC5/CCNB1/NEK2/ORC1/SKA1/KIF2C/RAD51AP1/AURKB/TOP2A/CDCA5/BUB1/TTK/CENPI/KNL1/CENPF/SKA3/CDK1/NDC80/RAD51/CDCA8/SNAI1/KIF18A/SPC24/CDT1/MCM4/SPC25/ESCO2/SGO2/ZWINT/BLM/MCM2	40	2.976528	2.824713
Negative	GOBP_ION_TRANSMEMBRANE_TRANSPORT	UBASH3B/SLC41A2/SLC29A4/TSPAN13/RASGRF2/ATP8B2/KCNE4/EMB/FXYD3/CYB5A/ANO5/ADRB2/SLC15A2/CACNA2D2/DRD1/SLC5A1/FOLR1	41	-1.71115	-1.98689
	GOBP_INORGANIC_ION_TRANSMEMBRANE_TRANSPORT	FXYD3/CYB5A/ANO5/SLC15A2/CACNA2D2/DRD1/SLC5A1	28	-1.84283	-1.9216
	GOBP_MULTICELLULAR_ORGANISMAL_HOMEOSTASIS	HOXC10/LAMA2/TMEM119/LCA5/COMP/ADRB2/AQP4/ABAT/SFTPD/DRD1	28	-1.59616	-1.48229
	GOBP_VESICLE_MEDIATED_TRANSPORT	SELE/C3/SCRN1/GRIP1/ACKR2/PLA2G3/EPHA3/CYTH3/ADRB2/IL13RA2/SFTPD/DRD1/FOLR1	39	-1.9387	-1.68394
	GOBP_CATION_TRANSPORT	FXYD3/CYB5A/PLA2G3/ADRB2/ABAT/SLC15A2/CACNA2D2/DRD1/SLC5A1	39	-1.65714	-1.73656

	GOBP_ION_TRANSPORT	FXYD3/CYB5A/PLA2G3/PLA2G2A/ANO5/ADRB2/ABAT/SLC15A2/CACNA2D2/DRD1/SLC5A1/FOLR1	55	-1.59326	-1.70027
	GOCC_ENDOPLASMIC_RETICULUM	METTL7B/MATN3/OLFM1/MOXD1/C3/GRIP1/DHRS3/TMEM119/COL14A1/RNF128/CYB5A/BACE2/ERP27/RASD1/THBS4/PLA2G2A/ANO5/SFTPD/DUOX1/DRD1/FOLR1/AADAC/HSD17B6/CYP4B1	76	-1.85048	-1.75342
	GOBP_CELLULAR_LIPID_METABOLIC_PROCESS	C3/DHRS3/ANG/PLA2G3/PLA2G2A/PLPPR4/AADAC/CYP4B1	23	-1.67057	-1.7211
	GOBP_CATION_TRANSMEMBRANE_TRANSPORT	TCN1/F2R/UBASH3B/SLC41A2/SLC29A4/TSPAN13/RASGRF2/KCNE4/FXYD3/CYB5A/ADRB2/SLC15A2/CACNA2D2/DRD1/SLC5A1	31	-1.70514	-1.83087
	GOCC_SIDE_OF_MEMBRANE	CD28/PLEKHA4/SELE/ACKR2/CYTH3/BCAM/AQP4/IL13RA2/ITGA9/FOLR1	28	-1.7099	-1.58904
	GOBP_TRANSMEMBRANE_TRANSPORT	SLC29A4/TSPAN13/RASGRF2/ATP8B2/KCNE4/EMB/C3/FXYD3/CYB5A/ANO5/ADRB2/AQP4/SLC15A2/CACNA2D2/DRD1/SLC5A1/FOLR1	49	-1.58847	-1.83616
	GOCC_EXTERNAL_SIDE_OF_PLASMA_MEMBRANE	SELE/ACKR2/BCAM/AQP4/IL13RA2/ITGA9/FOLR1	22	-1.83161	-1.58044
	GOBP_LIPID_METABOLIC_PROCESS	DPEP1/C3/DHRS3/ANG/PLA2G3/HCAR1/PLA2G2A/PLPPR4/AADAC/VSIG2/HSD17B6/CYP4B1	31	-1.91892	-1.81574
	GOBP_CELL_CELL_ADHESION	AJUBA/CD28/BMP7/EMB/SELE/GLDN/COL14A1/COMP/SDK2/PCDHGA10/PRKCZ/THBS4/AQP4/ABAT/SFTPD/ITGA9	41	-1.7432	-1.60619
	GOBP_POSITIVE_REGULATION_OF_IMMUNE_SYSTEM_PROCESS	CD28/S100A14/PDGFD/C3/PLA2G3/PRKCZ/THBS4/IL13RA2/PGC	28	-1.63862	-1.71605
	GOCC_ORGANELLE_SUBCOMPARTMENT	METTL7B/MOXD1/GRIP1/DHRS3/TMEM119/PCSK5/CYB5A/BACE2/PLA2G2A/ANO5/SFTPD/DRD1/FOLR1/AADAC/CYP4B1	31	-1.96341	-1.97917
	GOCC_INTRINSIC_COMPONENT_OF_PLASMA_MEMBRANE	EMB/PTPRH/PTGER2/SELE/ITGBL1/FXYD3/ACKR2/PCDHGA10/EPHA3/ADRB2/ADGRD1/BCAM/AQP4/PLPPR4/SLC15A2/ITGA9/DUOX1/DRD1/SLC5A1/FOLR1/TSPAN8/VSIG2	84	-1.6632	-1.75614
	GOCC_NUCLEAR_OUTER_MEMBRANE_ENDOPLASMIC_RETICULUM_MEMBRANE_NETWORK	METTL7B/MOXD1/GRIP1/DHRS3/TMEM119/CYB5A/PLA2G2A/ANO5/SFTPD/DRD1/FOLR1/AADAC/CYP4B1	23	-2.01964	-1.98658
	GOMF_INORGANIC_MOLECULAR_ENTITY_TRANSMEMBRANE_TRANSPORTER_ACTIVITY	FXYD3/CYB5A/ANO5/AQP4/SLC15A2/CACNA2D2/SLC5A1	21	-2.07911	-1.84285
	GOCC_ENDOSOME	LAMP5/RNF128/ARRDC4/ACKR2/PLA2G3/BACE2/EPHA3/PRKCZ/ADRB2/AQP4/SFTPD/SLC5A1/FOLR1/HSD17B6	32	-2.14942	-2.01621

Supplementary Table S14. A summary of the 23 hub genes identified in green and yellow modules.

Hub genes	RPG training set		TCGA validation set		CPTAC validation set		Pathways
	MM	GS	MM	GS	MM	GS	
ANLN	0.88	0.24	0.89	0.37	0.93	0.49	GOBP_POSITIVE_REGULATION_OF_CELLULAR_COMPONENT_BIOGENESIS/GOBP_ACTIN_FILAMENT_BASED_PROCESS/GOBP_POSITIVE_REGULATION_OF_CELLULAR_COMPONENT_ORGANIZATION/GOMF_ACTIN_BINDING/GOBP_LOCOMOTION
ASPM	0.88	0.44	0.9	0.37	0.93	0.48	GOBP_MAINTENANCE_OF_LOCATION_IN_CELL/GOBP_MEIOTIC_CELL_CYCLE_PROCESS/GOCC_MICROTUBULE_CYTOSKELETON/GOBP_MAINTENANCE_OF_LOCATION/GOBP_REGULATION_OF_NEUROBLAST_PROLIFERATION
BIRC5	0.87	0.34	0.9	0.38	0.96	0.53	GOBP_SENSORY_PERCEPTION_OF_MECHANICAL_STIMULUS/REACTOME_TP53_REGULATES_TRANSCRIPTION_OF_SEVERAL_ADDITIONAL_CELL_DEATH_GENES_WHOSE_SPECIFIC_ROLES_IN_P53_DEPENDENT_APOPTOSIS_REMAIN_UNCERTAIN/GOBP_REGULATION_OF_PEPTIDASE_ACTIVITY/REACTOME_INTERLEUKIN_4_AND_INTERLEUKIN_13_SIGNALING
BUB1	0.91	0.33	0.94	0.39	0.94	0.47	WP_ATM_SIGNALING_IN_DEVELOPMENT_AND_DISEASE/GOMF_PROTEIN_SERINE_THREONINE_TYROSINE_KINASE_ACTIVITY/REACTOME_M_PHASE/GOBP_CELL_DIVISION/GOBP_MITOTIC_SISTER_CHROMATID_SEGREGATION
BUB1B	0.85	0.37	0.93	0.39	0.95	0.5	REACTOME_RHO_GTPASES_ACTIVATE_FORMINS/GOBP_NEGATIVE_REGULATION_OF_NUCLEAR_DIVISION/GOCC_SPINDLE/REACTOME_APC_C_MEDIATED_DEGRADATION_OF_CELL_CYCLE_PROTEINS/GOBP_CELL_DIVISION/GOCC_NUCLEAR_UBIQUITIN_LIGASE_COMPLEX
CCN4	0.87	0.33	0.86	0.21	0.86	0.4	GOMF_PROTEIN_CONTAINING_COMPLEX_BINDING/GOBP_SMOOTH_MUSCLE_CELL_PROLIFERATION/GOMF_GROWTH_FACTOR_BINDING/GOBP_POSITIVE_REGULATION_OF_CELL_POPULATION_PROLIFERATION/GOBP_REGULATION_OF_MULTICELLULAR_ORGANISMAL_DEVELOPMENT
CDC20	0.8	0.31	0.92	0.39	0.94	0.51	GOBP_ANAPHASE_PROMOTING_COMPLEX_DEPENDENT_CATABOLIC_PROCESS/HALLMARK_MYC_TARGETS_V1/GOBP_CELLULAR_PROTEIN_CATABOLIC_PROCESS/PID_PLK1_PATHWAY/GOBP_POSITIVE_REGULATION_OF_PROTEOLYSIS_INVOLVED_IN_CELLULAR_PROTEIN_CATABOLIC_PROCESS
CDC25C	0.86	0.35	0.88	0.38	0.92	0.49	GOMF_KINASE_BINDING/GOBP_REGULATION_OF_CELL_CYCLE_G2_M_PHASE_TRANSITION/GOCC_NUCLEAR_SPECK/WP_CELL_CYCLE/WP_MONOAMINE_TRANSPORT/GOBP_MEIOSIS_I_CELL_CYCLE_PROCESS/GOBP_PROTEIN_DEPHOSPHORYLATION/REACTOME_CELL_CYCLE_CHECKPOINTS/GOBP_DEPHOSPHORYLATION/GOBP_REGULATION_OF_MITOTIC_CELL_CYCLE
CENPE	0.84	0.51	0.91	0.38	0.94	0.5	GOBP_CHROMOSOME_SEGREGATION/GOCC_SPINDLE_MIDZONE/GOBP_CELL_CYCLE_PROCESS/GOCC_SPINDLE_MICROTUBULE/GOMF_CYTOSKELETAL_PROTEIN_BINDING
CENPF	0.82	0.25	0.88	0.32	0.95	0.48	GOBP_CELL_CYCLE/GOBP_NEGATIVE_REGULATION_OF_CELLULAR_COMPONENT_ORGANIZATION/GOMF_CYTOSKELETAL_PROTEIN_BINDING/GOBP_MUSCLE_ORGAN_DEVELOPMENT/GOBP_REGULATION_OF_MITOTIC_SISTER_CHROMATID_SEGREGATION
CLSPN	0.83	0.38	0.91	0.32	0.94	0.49	GOBP_REGULATION_OF_CELL_CYCLE_PHASE_TRANSITION/GOBP_POSITIVE_REGULATION_OF_TRANSFERASE_ACTIVITY/REACTOME_CELL_CYCLE/WP_DNA_IRDAMAGE_AND_CELLULAR_RESPONSE_VIA_ATR/GOBP_POSITIVE_REGULATION_OF_PROTEIN_KINASE_ACTIVITY
COL5A2	0.93	0.26	0.93	0.22	0.93	0.42	GOBP_SENSORY_ORGAN_MORPHOGENESIS/REACTOME_NCAM1_INTERACTIONS/GOBP_SKELETAL_SYSTEM_DEVELOPMENT/REACTOME_NCAM_SIGNALING_FOR_NEURITE_OUT_GROWTH/GOBP_CELLULAR_RESPONSE_TO_ENDOGENOUS_STIMULUS
IQGAP3	0.83	0.44	0.83	0.29	0.89	0.47	GOBP_RAS_PROTEIN_SIGNAL_TRANSDUCTION/GOBP_REGULATION_OF_PROTEIN_PHOSPHORYLATION/PID_CDC42_PATHWAY/GOBP_GLAND_DEVELOPMENT/REACTOME_RHOA_GTPASE_CYCLE
KIF14	0.8	0.22	0.91	0.36	0.95	0.51	GOBP_CEREBELLAR_CORTEX_DEVELOPMENT/GOMF_ADENYL_NUCLEOTIDE_BINDING/REACTOME_RND1_GTPASE_CYCLE/GOBP_HINDBRAIN_DEVELOPMENT/GOBP_REGULATION_OF_CELL_CYCLE_PHASE_TRANSITION

KIF18B	0.81	0.38	0.92	0.34	0.96	0.53	GOCC_MITOTIC_SPINDLE_ASTRAL_MICROTUBULE/REACTOME_COPI_DEPENDENT_GOLGI_TO_ER_RETROGRADE_TRAFFIC/GOCC_MITOTIC_SPINDLE_MIDZONE/GOCC_SUPRAMOLECULAR_POLYMER/GOMF_ADENYL_NUCLEOTIDE_BINDING
KIF2C	0.88	0.48	0.95	0.36	0.96	0.52	REACTOME_INTRA_GOLGI_AND_RETROGRADE_GOLGI_TO_ER_TRAFFIC/REACTOME_M_PHASE/GOMF_SEQUENCE_SPECIFIC_DNA_BINDING/HALLMARK_SPERMATOGENESIS/GOCC_SUPRAMOLECULAR_COMPLEX
KNL1	0.88	0.44	0.88	0.37	0.93	0.5	GOBP_ENDOMEMBRANE_SYSTEM_ORGANIZATION/GOBP_GERM_CELL_DEVELOPMENT/GOBP_CELL_CYCLE/GOBP_CELLULAR_PROCESS_INVOLVED_IN_REPRODUCTION_IN_MULTICELLULAR_ORGANISM/REACTOME_SEPARATION_OF_SISTER_CHROMATIDS
MYBL2	0.85	0.29	0.9	0.34	0.94	0.55	GOBP_SPINDLE_ASSEMBLY/PID_E2F_PATHWAY/GOBP_MICROTUBULE_CYTOSKELETON_ORGANIZATION_INVOLVED_IN_MITOSIS/REACTOME_TRANSCRIPTIONAL_REGULATION_BY_THE_AP_2_TFAP2_FAMILY_OF_TRANSCRIPTION_FACTORS/GOBP_POSITIVE_REGULATION_OF_NUCLEOBASE_CONTAINING_COMPOUND_METABOLIC_PROCESS
NEK2	0.82	0.37	0.92	0.36	0.93	0.47	GOBP_REGULATION_OF_DNA_BIOSYNTHETIC_PROCESS/GOBP_MICROTUBULE_ORGANIZING_CENTER_ORGANIZATION/REACTOME_M_PHASE/REACTOME_MITOTIC_PROMETAPHASE/HALLMARK_SPERMATOGENESIS
RRM2	0.87	0.25	0.92	0.4	0.95	0.52	GOBP_DEOXYRIBOSE_PHOSPHATE_METABOLIC_PROCESS/KEGG_PYRIMIDINE_METABOLISM/GOBP_REGULATION_OF_CELL_CYCLE/GOBP_REGULATION_OF_MITOTIC_CELL_CYCLE_PHASE_TRANSITION/GOBP_DEOXYRIBONUCLEOTIDE_BIOSYNTHETIC_PROCESS
STIL	0.83	0.35	0.89	0.29	0.89	0.47	GOBP_EMBRYO_DEVELOPMENT_ENDING_IN_BIRTH_OR_EGG_HATCHING/GOBP_ORGANELLE_ASSEMBLY/GOBP_POSITIVE_REGULATION_OF_CELL_CYCLE_G1_S_PHASE_TRANSITION/GOBP_EMBRYONIC_ORGAN_DEVELOPMENT/GOBP_REGULATION_OF_ORGANELLE_ORGANIZATION
TOP2A	0.87	0.3	0.93	0.36	0.96	0.49	GOMF_RIBONUCLEOTIDE_BINDING/GOMF_ISOMERASE_ACTIVITY/GOMF_RNA_BINDING/GOBP_NEGATIVE_REGULATION_OF_CELLULAR_COMPONENT_ORGANIZATION/GOMF_DNA_BINDING_BENDING
UHRF1	0.88	0.43	0.88	0.34	0.9	0.54	GOBP_POSITIVE_REGULATION_OF_RNA_METABOLIC_PROCESS/GOMF_UBIQUITIN_LIKE_PROTEIN_TRANSFERASE_ACTIVITY/GOBP_POSITIVE_REGULATION_OF_MOLECULAR_FUNCTION/GOMF_CIS_REGULATORY_REGION_SEQUENCE_SPECIFIC_DNA_BINDING/GOMF_ZINC_ION_BINDING

The biologic annotations of these genes were listed as cancer hallmarks. The module membership and gene significance of the hub genes in RPG training set, TCGA validation set, and CPTAC validation set were listed. The biologic pathways involved were also shown for each hub gene. MM, module membership; GS, gene significance.

Supplementary Table S15. A summary of top five biologic pathways most significantly associated with each selected radiopathomics features.

Biologic functions	Feature	Category	Biologic pathways	r_value	FDR	Database
Signal_Transduction	DL PF_11	DL PF	GOBP_ACTIVATION_OF_ADENYLATE_CYCLASE_ACTIVITY	-0.46291	0.005107	GO
			GOMF_CATECHOLAMINE_BINDING	-0.46291	0.005107	GO
			REACTOME_COPI_DEPENDENT_GOLGI_TO_ER_RETROGRADE_TRAFFIC	0.425743	0.010776	REACTOME
			REACTOME_FACTORS_INVOLVED_IN_MEGAKARYOCYTE_DEVELOPMENT_AND_PLATELET_PRODUCTION	0.438851	0.008357	REACTOME
			REACTOME_GOLGI_TO_ER_RETROGRADE_TRANSPORT	0.425743	0.010776	REACTOME
	DL PF_7	DL PF	GOBP_NEGATIVE_REGULATION_OF_BONE_REMODELING	0.451923	0.006423	GO
			GOBP_NEGATIVE_REGULATION_OF_TISSUE_REMODELING	0.451923	0.006423	GO
			GOBP_PATHWAY_RESTRICTED_SMAD_PROTEIN_PHOSPHORYLATION	0.441979	0.007854	GO
			GOBP_POSITIVE_REGULATION_OF_RECEPTOR_MEDIATED_ENDOCYTOSIS	0.471732	0.004226	GO
			GOBP_TRABECULA_FORMATION	0.430448	0.009847	GO
	DL PF_8	DL PF	GOBP_ADENYLATE_CYCLASE_ACTIVATING_ADRENERGIC_RECEPTOR_SIGNALING_PATHWAY	-0.44697	0.007105	GO
			GOBP_ADRENERGIC_RECEPTOR_SIGNALING_PATHWAY	-0.44697	0.007105	GO
			REACTOME_AMINE_LIGAND_BINDING_RECEPTORS	-0.45994	0.005437	REACTOME
			WP_ALTERNATIVE_PATHWAY_OF_FETAL_ANDROGEN_SYNTHESIS	-0.46382	0.00501	WIKIPATHW
			WP_MONOAMINE_GPCRS	-0.45994	0.005437	WIKIPATHW
	DL RF_1	DL RF	GOBP_MICROTUBULE_DEPOLYMERIZATION	0.420129	0.01198	GO
			GOBP_PROTEIN_DEPOLYMERIZATION	0.378328	0.025029	GO
			GOBP_TRANSFORMING_GROWTH_FACTOR_BETA_RECEPTOR_SIGNALING_PATHWAY	-0.36754	0.029847	GO
			HALLMARK_SPERMATOGENESIS	0.366278	0.030459	HALLMARK
			WP_CORI_CYCLE	0.423009	0.011349	WIKIPATHW
	DL RF_10	DL RF	GOBP_CELLULAR_RESPONSE_TO_ABIOTIC_STIMULUS	-0.40739	0.01514	GO
			GOBP_NEGATIVE_REGULATION_OF_TRANSFORMING_GROWTH_FACTOR_BETA_RECEPTOR_SIGNALING_PATHWAY	0.426906	0.010539	GO
			GOBP_NEGATIVE_REGULATION_OF_TRANSMEMBRANE_RECEPTOR_PROTEIN_SERINE_THREONINE_KINASE_SIGNALING_PATHWAY	0.434956	0.009022	GO
			GOBP_REGULATION_OF_SMALL_GTPASE_MEDIATED_SIGNAL_TRANSDUCTION	-0.53283	0.000983	GO
			GOBP_RESPONSE_TO_TRANSFORMING_GROWTH_FACTOR_BETA	0.428611	0.010201	GO
	DL RF_2	DL RF	GOBP_RENAL_SYSTEM_PROCESS	-0.54027	0.000807	GO
			GOMF_EXTRACELLULAR_MATRIX_BINDING	-0.51746	0.001456	GO
			GOMF_HELICASE_ACTIVITY	0.51166	0.00168	GO
			KEGG_CALCIUM_SIGNALING_PATHWAY	-0.52472	0.001212	KEGG
			REACTOME_AMINE_LIGAND_BINDING_RECEPTORS	-0.50802	0.001836	REACTOME
	DL RF_4	DL RF	GOBP_HEAT_GENERATION	0.380633	0.024087	GO
			GOBP_NEGATIVE_REGULATION_OF_TRANSFORMING_GROWTH_FACTOR_BETA_RECEPTOR_SIGNALING_PATHWAY	0.35439	0.036727	GO
			GOBP_POSITIVE_REGULATION_OF_MAP_KINASE_ACTIVITY	0.375094	0.026401	GO
			GOBP_REGULATION_OF_SMALL_GTPASE_MEDIATED_SIGNAL_TRANSDUCTION	-0.33281	0.050761	GO
			GOCC_PERICENTRIC_HETEROCHROMATIN	0.336929	0.047793	GO

	DLRF_9	DLRF	GOBP_ACTIVATION_OF_ADENYLATE_CYCLASE_ACTIVITY	-0.60518	0.000117	GO
			GOBP_ADENYLATE_CYCLASE_ACTIVATING_ADRENERGIC_RECEPTOR_SIGNALING_PATHWAY	-0.61384	8.79E-05	GO
			GOBP_ADRENERGIC_RECEPTOR_SIGNALING_PATHWAY	-0.61384	8.79E-05	GO
			GOMF_CATECHOLAMINE_BINDING	-0.60518	0.000117	GO
			REACTOME_AMINE_LIGAND_BINDING_RECEPTORS	-0.59113	0.000185	REACTOME
	HRF_3	HRF	GOBP_ADENYLATE_CYCLASE_ACTIVATING_ADRENERGIC_RECEPTOR_SIGNALING_PATHWAY	-0.45508	0.006018	GO
			GOBP_POSITIVE_REGULATION_OF_CELLULAR_EXTRAVASATION	0.471748	0.004224	GO
			REACTOME_SIGNALING_BY_PDGF	0.45928	0.005513	REACTOME
			WP_ALTERNATIVE_PATHWAY_OF_FETAL_ANDROGEN_SYNTHESIS	-0.48284	0.003305	WIKIPATHW
			WP_ANGIOTENSIN_II_RECEPTOR_TYPE_1_PATHWAY	0.46074	0.005347	WIKIPATHW
	HRF_4	HRF	BIOCARTA_MPR_PATHWAY	0.387998	0.021272	BIOCARTA
			GOBP_ACTIVATION_OF_ADENYLATE_CYCLASE_ACTIVITY	-0.37105	0.028205	GO
			GOBP_ADENYLATE_CYCLASE_ACTIVATING_ADRENERGIC_RECEPTOR_SIGNALING_PATHWAY	-0.42416	0.011105	GO
			GOBP_ADRENERGIC_RECEPTOR_SIGNALING_PATHWAY	-0.42416	0.011105	GO
			GOBP_REGULATION_OF_UBIQUITIN_PROTEIN_TRANSFERASE_ACTIVITY	0.389767	0.020638	GO
	HRF_5	HRF	GOBP_CELLULAR_RESPONSE_TO_IONIZING_RADIATION	0.364879	0.031148	GO
			GOBP_REGULATION_OF_RAS_PROTEIN_SIGNAL_TRANSDUCTION	0.326754	0.055383	GO
			GOBP_REGULATION_OF_SMALL_GTPASE_MEDIATED_SIGNAL_TRANSDUCTION	0.368382	0.029447	GO
			GOBP_RESPONSE_TO_DEXAMETHASONE	0.299729	0.08024	GO
			GOBP_RESPONSE_TO_IONIZING_RADIATION	0.336652	0.047988	GO
Metastasis_and_Invasion	DLPF_2	DLPF	GOBP_NEGATIVE_REGULATION_OF_BONE_REMODELING	0.398606	0.017703	GO
			GOBP_NEGATIVE_REGULATION_OF_TISSUE_REMODELING	0.398606	0.017703	GO
			GOBP_OSTEOBLAST_PROLIFERATION	0.418623	0.012321	GO
			GOBP_REGULATION_OF_OSTEOBLAST_PROLIFERATION	0.418623	0.012321	GO
			GOBP_TRABECULA_FORMATION	0.391036	0.020194	GO
	DLRF_7	DLRF	REACTOME_COPI_DEPENDENT_GOLGI_TO_ER_RETROGRADE_TRAFFIC	-0.56554	0.0004	REACTOME
			REACTOME_GOLGI_TO_ER_RETROGRADE_TRANSPORT	-0.56554	0.0004	REACTOME
			REACTOME_KINESINS	-0.56554	0.0004	REACTOME
			REACTOME_MHC_CLASS_II_ANTIGEN_PRESENTATION	-0.63023	4.96E-05	REACTOME
			REACTOME_MITOTIC_G2_G2_M_PHASES	-0.59146	0.000183	REACTOME
	HPF_4	HPF	GOBP_CELL_ADHESION_MEDIATED_BY_INTEGRIN	0.40837	0.014873	GO
			GOBP_CELLULAR_EXTRAVASATION	0.435446	0.008936	GO
			GOBP_OVULATION_CYCLE	0.435473	0.008931	GO
			GOBP_POSITIVE_REGULATION_OF_SMOOTH_MUSCLE_CELL_MIGRATION	0.43208	0.009541	GO
			GOBP_REGULATION_OF_CELLULAR_EXTRAVASATION	0.417307	0.012627	GO
Metabolic	DLPF_10	DLPF	REACTOME_COPI_DEPENDENT_GOLGI_TO_ER_RETROGRADE_TRAFFIC	0.367228	0.029999	REACTOME
			REACTOME_FACTORS_INVOLVED_IN_MEGAKARYOCYTE_DEVELOPMENT_AND_PLATELET_PRODUCTION	0.3786	0.024916	REACTOME
			REACTOME_GOLGI_TO_ER_RETROGRADE_TRANSPORT	0.367228	0.029999	REACTOME
			REACTOME_KINESINS	0.367228	0.029999	REACTOME
			REACTOME_MHC_CLASS_II_ANTIGEN_PRESENTATION	0.378252	0.02506	REACTOME
			GOBP_BONE_REMODELING	-0.52471	0.001212	GO

	DLPF_13	DLPF	GOBP_REGULATION_OF_BMP_SIGNALING_PATHWAY	-0.4856	0.003105	GO
			GOBP_REGULATION_OF_SISTER_CHROMATID_COHESION	0.524888	0.001207	GO
			GOMF_CYTOSKELETAL_MOTOR_ACTIVITY	0.482656	0.003319	GO
			WP_REGULATION_OF_SISTER_CHROMATID_SEPARATION_AT_THE_METAPHASEANAPHASE_TRANSITION	0.531829	0.001009	WIKIPATHW
	DLPF_9	DLPF	REACTOME_COPI_DEPENDENT_GOLGI_TO_ER_RETROGRADE_TRAFFIC	0.594183	0.000168	REACTOME
			REACTOME_FACTORS_INVOLVED_IN_MEGAKARYOCYTE_DEVELOPMENT_AND_PLATELET_PRODUCTION	0.605044	0.000118	REACTOME
			REACTOME_GOLGI_TO_ER_RETROGRADE_TRANSPORT	0.594183	0.000168	REACTOME
			REACTOME_KINESINS	0.594183	0.000168	REACTOME
			REACTOME_MHC_CLASS_II_ANTIGEN_PRESENTATION	0.647191	2.65E-05	REACTOME
	HPF_2	HPF	GOBP_DNA_BIOSYNTHETIC_PROCESS	0.492961	0.002623	GO
			GOBP_VASCULAR_PROCESS_IN_CIRCULATORY_SYSTEM	-0.53057	0.001043	GO
			GOMF_ATP_HYDROLYSIS_ACTIVITY	0.518163	0.00143	GO
			GOMF_DNA_SECONDARY_STRUCTURE_BINDING	0.515967	0.001511	GO
			REACTOME_AMINE_LIGAND_BINDING_RECEPTORS	-0.48567	0.003101	REACTOME
DNA_Damage_and_Repair	DLPF_12	DLPF	GOBP_DNA_RECOMBINATION	0.534561	0.000939	GO
			GOBP_MEIOSIS_I_CELL_CYCLE_PROCESS	0.515061	0.001545	GO
			GOBP_MEIOTIC_CELL_CYCLE	0.50016	0.002216	GO
			GOBP_REGULATION_OF_UBIQUITIN_PROTEIN_TRANSFERASE_ACTIVITY	0.535604	0.000914	GO
			HALLMARK_G2M_CHECKPOINT	0.522042	0.001297	HALLMARK
	DLPF_3	DLPF	GOBP_DOUBLE_STRAND_BREAK_REPAIR_VIA_BREAK_INDUCED_REPLICATION	-0.33345	0.050286	GO
			GOBP_REGULATION_OF_INSULIN_LIKE_GROWTH_FACTOR_RECEPTOR_SIGNALING_PATHWAY	0.327016	0.055177	GO
			GOMF_DNA_REPLICATION_ORIGIN_BINDING	-0.31931	0.061519	GO
			REACTOME_FACTORS_INVOLVED_IN_MEGAKARYOCYTE_DEVELOPMENT_AND_PLATELET_PRODUCTION	0.304255	0.075564	REACTOME
			REACTOME_MHC_CLASS_II_ANTIGEN_PRESENTATION	0.311427	0.068591	REACTOME
	DLRF_11	DLRF	GOBP_DNA_BIOSYNTHETIC_PROCESS	0.510657	0.001722	GO
			GOBP_DNA_UNWINDING_INVOLVED_IN_DNA_REPLICATION	0.495727	0.00246	GO
			GOBP_HOMOLOGOUS_RECOMBINATION	0.487504	0.002974	GO
			GOBP_POSITIVE_REGULATION_OF_DNA_METABOLIC_PROCESS	0.530228	0.001052	GO
			GOMF_ATP_HYDROLYSIS_ACTIVITY	0.487823	0.002952	GO
	DLRF_5	DLRF	GOBP_DOUBLE_STRAND_BREAK_REPAIR_VIA_BREAK_INDUCED_REPLICATION	-0.43871	0.00838	GO
			GOCC_DNA_REPLICATION_PREINITIATION_COMPLEX	-0.37981	0.024419	GO
			GOMF_DNA_REPLICATION_ORIGIN_BINDING	-0.40541	0.015687	GO
			REACTOME_DNA_STRAND_ELONGATION	-0.37981	0.024419	REACTOME
			WP_ALTERNATIVE_PATHWAY_OF_FETAL_ANDROGEN_SYNTHESIS	0.40541	0.015688	WIKIPATHW
	HRF_2	HRF	GOBP_ADENYLATE_CYCLASE_ACTIVATING_G_PROTEIN_COUPLED_RECEPTOR_SIGNALING_PATHWAY	0.495668	0.002463	GO
			GOBP_DNA_BIOSYNTHETIC_PROCESS	-0.53439	0.000944	GO
			GOMF_HELICASE_ACTIVITY	-0.49731	0.00237	GO
			WP_ALTERNATIVE_PATHWAY_OF_FETAL_ANDROGEN_SYNTHESIS	0.491574	0.002709	WIKIPATHW
			WP_CORI_CYCLE	-0.56333	0.000426	WIKIPATHW
Cell_Proliferation_and_Growth	DLPF_14	DLPF	GOBP_ESTABLISHMENT_OF_MITOTIC_SPINDLE_LOCALIZATION	0.535244	0.000923	GO
			GOBP_SPINDLE_LOCALIZATION	0.560419	0.000463	GO
			GOCC_KINESIN_COMPLEX	0.529654	0.001068	GO
			GOMF_MICROTUBULE_MOTOR_ACTIVITY	0.52585	0.001178	GO

			REACTOME_COPI_DEPENDENT_GOLGI_TO_ER_RETROGRADE_TRAFFIC	0.519119	0.001396	REACTOME
	DLPF_4	DLPF	GOBP_HEAT_GENERATION	-0.40165	0.016777	GO
			GOBP_POSITIVE_REGULATION_OF_NERVOUS_SYSTEM_DEVELOPMENT	0.386183	0.021939	GO
			GOBP_REGULATION_OF_OSSIFICATION	-0.45382	0.006176	GO
			PID_INTEGRIN3_PATHWAY	0.38296	0.023166	PID
			WP_ALTERNATIVE_PATHWAY_OF_FETAL_ANDROGEN_SYNTHESIS	-0.46198	0.005209	WIKIPATHW
	DLRF_6	DLRF	GOBP_DNA_BIOSYNTHETIC_PROCESS	-0.37165	0.027929	GO
			GOBP_GANGLION_DEVELOPMENT	-0.35918	0.034084	GO
			GOBP_POSITIVE_REGULATION_OF_DNA_METABOLIC_PROCESS	-0.35243	0.037857	GO
			GOBP_PROTEIN_DEPOLYMERIZATION	-0.35051	0.038983	GO
			KEGG_P53_SIGNALING_PATHWAY	-0.39958	0.017403	KEGG
	DLRF_8	DLRF	GOBP_MITOTIC_G2_DNA_DAMAGE_CHECKPOINT_SIGNALING	-0.42253	0.011452	GO
			GOBP_NEGATIVE_REGULATION_OF_MITOTIC_CELL_CYCLE	-0.42197	0.011573	GO
			GOBP_SIGNAL_TRANSDUCTION_IN_RESPONSE_TO_DNA_DAMAGE	-0.46338	0.005056	GO
			REACTOME_HDR_THROUGH_SINGLE_STRAND_ANNEALING_SSA	-0.41229	0.01385	REACTOME
			REACTOME_REGULATION_OF_TP53_ACTIVITY_THROUGH_PHOSPHORYLATION	-0.42759	0.010402	REACTOME
Cell_Differentiation	DLPF_1	DLPF	GOBP_APPENDAGE_DEVELOPMENT	-0.34479	0.042519	GO
			GOBP_CAMERA_TYPE_EYE_MORPHOGENESIS	-0.33688	0.047824	GO
			GOBP_POSITIVE_REGULATION_OF_CELL_CELL_ADHESION	-0.35481	0.03649	GO
			REACTOME_FACTORS_INVOLVED_IN_MEGAKARYOCYTE_DEVELOPMENT_AND_PLATELET_PRODUCTION	0.354992	0.036386	REACTOME
			REACTOME_MHC_CLASS_II_ANTIGEN_PRESENTATION	0.37547	0.026238	REACTOME
	DLPF_5	DLPF	GOBP_NEGATIVE_REGULATION_OF_CARTILAGE_DEVELOPMENT	0.47179	0.00422	GO
			GOBP_NEGATIVE_REGULATION_OF_CHONDROCYTE_DIFFERENTIATION	0.399343	0.017474	GO
			GOBP_POSITIVE_REGULATION_OF_VASCULATURE_DEVELOPMENT	0.427386	0.010443	GO
			GOBP_REGULATION_OF_CARTILAGE_DEVELOPMENT	0.443657	0.007595	GO
			GOBP_RESPONSE_TO_XENOBIOTIC_STIMULUS	0.389172	0.02085	GO
	DLPF_6	DLPF	GOBP_NEGATIVE_REGULATION_OF_CARTILAGE_DEVELOPMENT	0.453364	0.006235	GO
			GOBP_POSITIVE_REGULATION_OF_VASCULATURE_DEVELOPMENT	0.41358	0.013526	GO
			GOBP_REGULATION_OF_CARTILAGE_DEVELOPMENT	0.37418	0.0268	GO
			GOBP_TISSUE_REMODELING	0.320888	0.060176	GO
			WP_CANONICAL_AND_NONCANONICAL_TGFB_SIGNALING	0.354928	0.036422	WIKIPATHW
	DLRF_3	DLRF	GOBP_FOREBRAIN_GENERATION_OF_NEURONS	-0.36878	0.029258	GO
			GOBP_NERVE_DEVELOPMENT	-0.38307	0.023124	GO
			GOBP_NUCLEOSOME_ORGANIZATION	0.380399	0.024181	GO
			GOBP_TELENCEPHALON_DEVELOPMENT	-0.38413	0.022714	GO
			REACTOME_CONDENSATION_OF_PROPHASE_CHROMOSOMES	0.39444	0.019039	REACTOME
	HPF_1	HPF	BIOCARTA_MPR_PATHWAY	0.297341	0.082796	BIOCARTA
			GOBP_ARTERY_MORPHOGENESIS	-0.30453	0.075288	GO
			GOBP_POSITIVE_REGULATION_OF_NEUROGENESIS	-0.37064	0.028391	GO
			GOCC_CYTOPLASMIC_MICROTUBULE	-0.29484	0.085544	GO
			GOMF_HORMONE_ACTIVITY	-0.29572	0.084566	GO
	HPF_3	HPF	GOBP_MUSCLE_TISSUE_DEVELOPMENT	0.43324	0.009329	GO
			GOBP_NEURON_PROJECTION_GUIDANCE	0.430658	0.009807	GO
			GOBP_POSITIVE_REGULATION_OF_NERVOUS_SYSTEM_DEVELOPMENT	0.445606	0.007303	GO

			GOBP_TELENCEPHALON_DEVELOPMENT	0.529954	0.001059	GO
			WP_PLATELETMEDIATED_INTERACTIONS_WITH_VASCULAR_AND_CIRCULATING_CELLS	0.455452	0.005972	WIKIPATHW
	HPF_5	HPF	GOBP_NEURON_MIGRATION	-0.4931	0.002615	GO
			GOBP_POSITIVE_REGULATION_OF_NEUROGENESIS	-0.37216	0.027698	GO
			GOBP_TELENCEPHALON_DEVELOPMENT	-0.38544	0.022219	GO
			HALLMARK_GLYCOLYSIS	-0.38096	0.023957	HALLMARK
			REACTOME_MET_ACTIVATES_PTK2_SIGNALING	-0.36945	0.028942	REACTOME
	HRF_1	HRF	GOBP_MICROTUBULE_DEPOLYMERIZATION	-0.45509	0.006016	GO
			GOBP_OUTFLOW_TRACT_MORPHOGENESIS	0.53339	0.000969	GO
			GOBP_PROTEIN_DEPOLYMERIZATION	-0.44883	0.006842	GO
			GOBP_REGULATION_OF_HORMONE_LEVELS	0.447655	0.007007	GO
			GOBP_REGULATION_OF_UBIQUITIN_PROTEIN_TRANSFERASE_ACTIVITY	-0.4684	0.004542	GO

Based on their associated pathways, the selected radiopathomics features were categorized into six biological functions.

Supplementary Table S16. Minimum detectable correlation coefficient (MDCC) for each set at 80% statistical power (α = 0.05).

Dataset	Sample Size (n)	Minimum Detectable Correlation (α = 0.05, power = 0.8)
RPG training set	36	0.45
RPG-TCGA validation set	18	0.58
RPG-CPTAC validation set	24	0.52

The results indicate that the sample sizes in this study are sufficient to robustly identify correlations of moderate effect size and larger (where $|r| > 0.4$-0.5 is typically considered moderate). However, there may be limited power to detect weaker correlations ($|r| < 0.3$-0.4), which provides crucial context for interpreting null associations.
